# Sensitivity of Atmospheric River Vapor Transport and Precipitation to Uniform Sea Surface Temperature Increases

**DOI:** 10.1029/2020JD033421

**Published:** 2020-10-29

**Authors:** Elizabeth E. McClenny, Paul A. Ullrich, Richard Grotjahn

**Affiliations:** ^1^ Atmospheric Science University of California, Davis Davis CA USA

**Keywords:** atmospheric rivers, CC scaling, sensitivity, aquaplanet, attribution

## Abstract

Filaments of intense vapor transport called atmospheric rivers (ARs) are responsible for the majority of poleward vapor transport in the midlatitudes. Despite their importance to the hydrologic cycle, there remain many unanswered questions about changes to ARs in a warming climate. In this study we perform a series of escalating uniform SST increases (+2, +4, and +6K, respectively) in the Community Atmosphere Model version 5 in an aquaplanet configuration to evaluate the thermodynamic and dynamical response of AR vapor content, transport, and precipitation to warming SSTs. We find that AR column integrated water vapor (IWV) is especially sensitive to SST and increases by 6.3–9.7% per degree warming despite decreasing relative humidity through much of the column. Further analysis provides a more nuanced view of AR IWV changes: Since SST warming is modest compared to that in the midtroposphere, computing fractional changes in IWV with respect to SST results in finding spuriously large increases. Meanwhile, results here show that AR IWV transport increases relatively uniformly with temperature and at consistently lower rates than IWV, as modulated by systematically decreasing low‐level wind speeds. Similarly, changes in AR precipitation are related to a compensatory relationship between enhanced near‐surface moisture and damped vertical motions.

## Key Points

- Aquaplanet atmospheric rivers grow in size and intensity as sea surface temperatures increase
- Increases in column water vapor beyond Clausius‐Clapeyron rates mask drying at upper levels
- Weakening dynamics damp changes in atmospheric river vapor transport and precipitation

## 1 Introduction

Atmospheric rivers (ARs) are shallow (up to 4 km deep), filamentary (<1,000 km wide, ∼2,000 km long) streams of concentrated vapor typically found in the extratropics and midlatitudes (Cordeira et al., 2013; Ralph et al., 2004, 2018). While ARs occupy only ∼10% of the Earth's zonal circumference in the midlatitudes, they perform virtually all of the meridional vapor transport outside of the tropics (Ralph et al., 2004; Zhu & Newell, 1998). When AR vapor is lifted—for instance, by mountain barriers or the warm conveyor belt of an extratropical cyclone (ETC)—they can produce substantial precipitation. According to a study performed by Paltan et al. (2017), ARs alone are responsible for 22*%* of annual total global runoff. As prominent features of the global hydrological cycle, the variability of ARs has important consequences for global energy balance and regional water resources. In particular, understanding the response of AR precipitation to increasing surface temperatures is a significant open topic today.

The Clausius‐Clapeyron (CC) relation predicts a ∼6–7% increase in saturation vapor pressure (*e*^∗^) near the surface for each kelvin increase in surface temperature (Trenberth et al., 2003). While this figure only applies to local changes, global‐mean column‐integrated water vapor (IWV) nevertheless increases at approximately CC rates (Held & Soden, 2006; O'Gorman & Muller, 2010), though significant regional variations exist (O'Gorman & Schneider, 2009a). Although mean state changes may not hold for individual weather events, AR IWV has increased at rates comparable to that predicted by CC, in turn resulting in greater integrated vapor transport (IVT) in studies comparing present‐day (PD) and Representative Concentration Pathway (RCP) 8.5 end‐of‐century (EOC) conditions (Payne & Magnusdottir, 2015; Shields & Kiehl, 2016; Warner et al., 2014). Gao et al. (2015) explicitly tested the enhancement of AR IWV with respect to near‐surface temperatures by isolating the thermodynamic and dynamical contributions to AR IVT under PD and RCP8.5 conditions and found that AR IWV followed a “super‐CC” increase. In other words, the CC relation appeared to underpredict the change in AR IWV compared to that actually measured, which Gao et al. (2015) attributed to AR vapor originating from a warmer ocean basin that was not included in their calculations. However, it has since been argued that such “super‐CC” increases in AR IWV are consistent with the approximately saturated, moist‐neutral conditions characteristic of strong ARs (Ralph et al., 2017). Specifically, latent heat release aloft enhances upper‐tropospheric warming with respect to surface warming, thereby driving AR IWV increases beyond CC predictions conditioned on surface temperatures (Payne et al., 2020). Such a result has been noted previously in zonal mean IWV studies (O'Gorman & Schneider, 2009a, 2009b), as well as orographic precipitation studies (Siler & Roe, 2014), but not strictly tested with respect to ARs.

Global‐mean precipitation rates will increase at lower fractional rates than global‐mean IWV due to energetic constraints (2–3% per K surface warming Held & Soden, 2006; O'Gorman & Muller, 2010; O'Gorman et al., 2012); likewise, theoretical considerations for AR precipitation rates also predict lower fractional enhancement than for AR IWV. Since strong ARs are approximately moist‐neutral (Ralph et al., 2017), it is expected that their internal vertical velocities will not change appreciably as the climate warms (O'Gorman, 2015); given this, it can be shown that extreme, nonorographic AR precipitation rates will increase fractionally at the rate of near‐surface *e*^∗^, which is lower than that for AR IWV (Payne et al., 2020). Since orographic enhancement is a frequent driver of extreme AR precipitation (e.g., Ralph et al., 2006), the previously described changes in the vertical structure of atmospheric moisture have additional consequences. Specifically, the amplified warming aloft also drives the largest fractional increases in condensation higher in the column, in turn limiting moisture availability for precipitation on the windward side: Siler and Roe (2014) found that orographically enhanced precipitation increased at larger rates on the leeward side of a mountain range than upsteam of the crest (12.2% vs. 8.8%). Another study comparing historical and RCP8.5 ARs in a 29‐member ensemble found a 35% increase in the number of AR days but only a 28% increase in AR days associated with extreme precipitation (Hagos et al., 2016). Last, Warner et al. (2014) examined U.S. west coast ARs in a 10‐member model ensemble under historical and RCP8.5 conditions and found that AR IVT increased by 25–30% while offshore AR precipitation rates increased by 15–39%, though they noted that uneven surface warming may have contributed to the ranges of the calculations.

Theory‐based predictions for AR moisture transport and precipitation provide excellent physical context but can be difficult to test in “comprehensive climate models” (here defined as those which contain sea ice, land, and an interactive ocean) due to the sheer complexity of their many interacting components (Held, 2005). Therefore, we suggest that a simplified experiment design will help elucidate the thermodynamic and dynamical drivers of AR moisture transport and precipitation. Specifically, we suggest that the use of an aquaplanet (AQP) model—a water‐covered world without land, sea ice, interactive ocean, or topography (Neale & Hoskins, 2000)—is ideally suited for such a study. Previous studies have leveraged the simplified AQP framework to isolate the physical drivers of AR activity (Hagos et al., 2015; Swenson et al., 2018). Likewise, we use an AQP here to isolate SST forcing from other climate change effects (e.g., differential land‐sea warming) inherently present in comprehensive climate models, while the use of uniform SST warming can help us further rule out impacts from changing meridional SST gradients. Hence, we set out here to study the thermodynamically and dynamically driven changes of AR moisture transport and precipitation to a series of progressively larger uniform SST increases in an AQP model. Specifically, this research investigates the following questions:

- What are the thermodynamic responses of AR moisture transport and precipitation rates to a collection of uniform SST increases?
- How do dynamic quantities (e.g., midlatitude circulation and wind) respond to increasing SSTs?
- How do these dynamic quantities serve to enhance or damp changes in AR vapor transport and precipitation?

In all, this study seeks to contextualize AR climate change statistics—whether those established from observations or from studies in comprehensive climate models—by isolating the impacts of warming SSTs. We will not only test physical linkages between SST increases and AR statistics but will break down more complex AR processes (e.g., precipitation) into simple constituents (e.g., near‐surface specific humidity and vertical velocity) to further our understanding.

A secondary goal of this manuscript is the introduction of a simple and novel AR detection tool (ARDT) developed as part of the TempestExtremes framework (Ullrich & Zarzycki, 2017). Unlike other ARDTs in the literature (e.g., see  Shields et al., 2018) the proposed scheme relies on a threshold on the Laplacian of the IVT (rather than the IVT itself). This has the advantage of detecting AR structures as ridges in the IVT field regardless of changes to the background that occur in response to surface warming. An optional algorithm for filtering tropical cyclone objects is also described.

This paper is organized as follows: section 2 outlines methods, including model parameters and detection tools; section 3 presents relevant AR, non‐AR, and total (AR + non‐AR) statistics, including column‐integrated quantities as well as vertical profiles for all runs; finally, section 4 provides a brief summary of our findings, as well as concluding thoughts.

## 2 Methods

### 2.1 Model Setup

We use the Community Earth System Model (CESM) Version 2 with Community Atmosphere Model version 5 (CAM5) physics (Neale et al., 2012). The spectral element dynamical core is employed with NE60 horizontal resolution (0.5° average spacing between degrees of freedom along the equatorial band). Output is remapped to a uniform 0.5° finite volume grid using the TempestRemap software suite (Ullrich et al., 2016; Ullrich & Taylor, 2015) before calculating derived variables, detecting ARs, or performing analyses.

To isolate the influence of SST on AR precipitation, we run CAM in its AQP configuration, meaning it has no land, sea ice, or topography; it is a water‐covered world. We prescribe a data ocean in which SST thermally forces the atmosphere while remaining fixed. As a “Baseline” SST scenario, we use the “QOBS” profile from Neale and Hoskins (2000), while the warming scenarios feature +2K, +4K, and +6 K uniform SST increases over QOBS, respectively. This uniform increase includes polar regions: Where the original QOBS formulation sets SST poleward of 60° at 0°C, we set these points equal to the uniform SST increase to prevent effects associated with a changing meridional SST gradient.

All SST scenarios are symmetric about the equator and zonally uniform, solar radiation is fixed at perpetual‐equinox conditions, and there is no axial tilt. Carbon dioxide concentrations for all runs are set at a uniform 348 ppm (the default value for CAM5 AQP), aerosol cloud interactions are turned off, and the only aerosol emissions come from sea salt, which the model diagnoses from surface wind. The zonal and hemispheric homogeneity of the boundary conditions, along with the lack of seasonality, allows for many more ARs under the same boundary conditions and forcing, so the sample size reduces confidence bounds. Medeiros et al. (2016) found that 2‐year, zonal mean statistics for water vapor, horizontal and vertical winds, and precipitation were statistically robust with respect to a 16‐year total AQP run. Similarly, Yang et al. (2013) found that a single year provided stable statistics for zonal mean precipitation extremes in a five‐year AQP experiment. Testing of our own determined that zonal mean AR statistics converged within 18 months (supporting information Figure S1), so we maintain that the 30 months of statistics shown here sufficiently capture bulk AR properties. Last, insofar as the northern and southern hemispheres of an aquaplanet can be considered independent from one another, we average both hemispheres together and consider it as virtually doubling the sample size, leaving us with effectively 2*N* months of data for each SST scenario, where N=30.

### 2.2 AR Detection

The American Meteorological Society (AMS) definition for AR contains little quantitative guidance (AMS, 2019), allowing for flexibility in ARDT development but also contributing to differences across ARDTs. These differences, which include variations in terms of detection variable (e.g., IWV and IVT), thresholds on the intensity of detection variables, geometry, event persistence, and/or other detection considerations, can ultimately affect conclusions about AR characteristics and impacts (O'Brien et al., 2020; Payne et al., 2020; Rutz et al., 2019; Shields et al., 2018). While some ARDTs rely on relative moisture thresholds derived from climatology (e.g., Guan & Waliser, 2015; Lavers et al., 2012), others use absolute thresholds for either IWV (Ralph et al., 2004; Wick et al., 2013) or IVT (Equation 1) (Leung & Qian, 2009; Rutz et al., 2013). In general, ARDT authors tend to condition AR detection on IVT rather than IWV, in part because a pure IWV threshold does not capture the nature of ARs as midlatitude processes that transport moisture, as well as because IWV thresholds capture too much of the tropical moisture belt. We compute IVT as

(1) $$IVT=\sqrt{{\left(-\frac{1}{g}\int_{p_{0}}^{p_{T}}qudp\right)}^{2}+{\left(-\frac{1}{g}\int_{p_{0}}^{p_{T}}qvdp\right)}^{2}}$$

where $p_{0}=$ 1,000 hPa, $p_{T}=300$ hPa, *g* is gravitational acceleration, *q* is specific humidity, and *u* and *v* are the zonal and meridional wind velocities, respectively.

We use an original, objective ARDT which is available as part of the TempestExtremes (TE) software suite (Ullrich & Zarzycki, 2017; Zarzycki & Ullrich, 2017). TE uses the following criteria to detect AR conditions (i.e., the presence of an AR at an individual time step) at a latitude‐longitude grid point:

1. The grid point is poleward of 20°N/S.
2. The Laplacian of IVT at the grid point is <−40,000 kg m^−1^ s^−1^ rad^−2^. We compute this using a 9‐point discrete Laplacian with a stencil radius of approximately 800 km, about the mean AR width found both in an observational study by Ralph et al. (2017) as well as a reanalysis study by Guan et al. (2018).
3. The grid point is part of ≥50 connected grid points (an area of approximately 125,000 km^2^) which meet the above criteria, as determined via a simple floodfill algorithm. This area requirement removes pointwise enhancements in the IVT field which would otherwise be counted as ARs, and we found that no explicit length or width requirements beyond this were necessary to achieve a sampling of ARs with a characteristic filamentary shape.
4. The grid point does not belong to a tropical cyclone (see section 2.3 for details)

We emphasize here two important points about our ARDT. First, our use of an absolute threshold for the Laplacian of IVT, rather than for IVT itself, allows TE to act similarly to a “relative” ARDT (e.g., an ARDT conditioned on climatological IVT percentiles; see Rutz et al., 2019; Shields et al., 2018) and precludes any necessity to enforce different detection thresholds despite the different background climatology of each SST run. This is because the Laplacian identifies regions where IVT has increased to a ridge over a short distance (∼800 km) relative to the local background IVT. Offline sensitivity tests determined that the Laplacian threshold was sufficiently strong that it never captures points that do not satisfy the threshold of IVT >250 kg/m/s typically used for ARs (Ralph et al., 2019; Shields et al., 2018). Therefore, whenever this manuscript references ARs in the context of those detected by TE, it refers to any object whose IVT represents a local ridge in the field. We recognize that our detection criteria often result in the inclusion of tropical cyclones (TCs), so we filter them out with a separate detection criteria (see section 2.3 for details). Second, the Laplacian threshold, its radius, and the grid point number (area) requirement were all determined via manual inspection of detected objects. These thus represent tuning parameters in the ARDT and should not be taken as absolute. For reference, we included TE in the Atmospheric River Tracking Method Intercomparison Project (ARTMIP; see Rutz et al., 2019; Shields et al., 2018), which seeks to quantify how differing ARDTs result in differing AR statistics. Within these studies, TE tends to exhibit behavior close to the median for relevant AR statistics, relative to the entire ensemble of ARTMIP ARDTs (Rutz et al., 2019; Shields et al., 2018).

**Figure 1:**
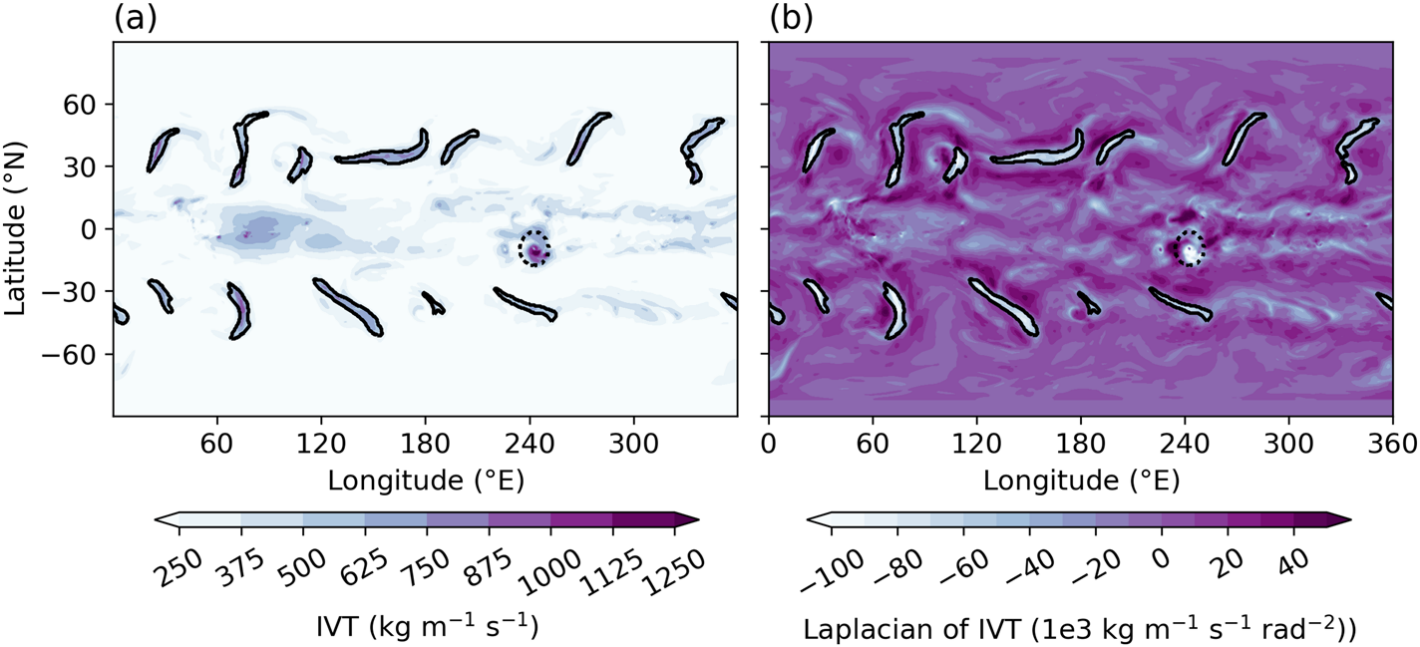
Snapshot of ARs (solid black outlines) as well as a TC (dotted black outlines) as identified by TE. We show them here on a field of (a) IVT and (b) the Laplacian of IVT from the Baseline SST run.

**Figure 2:**
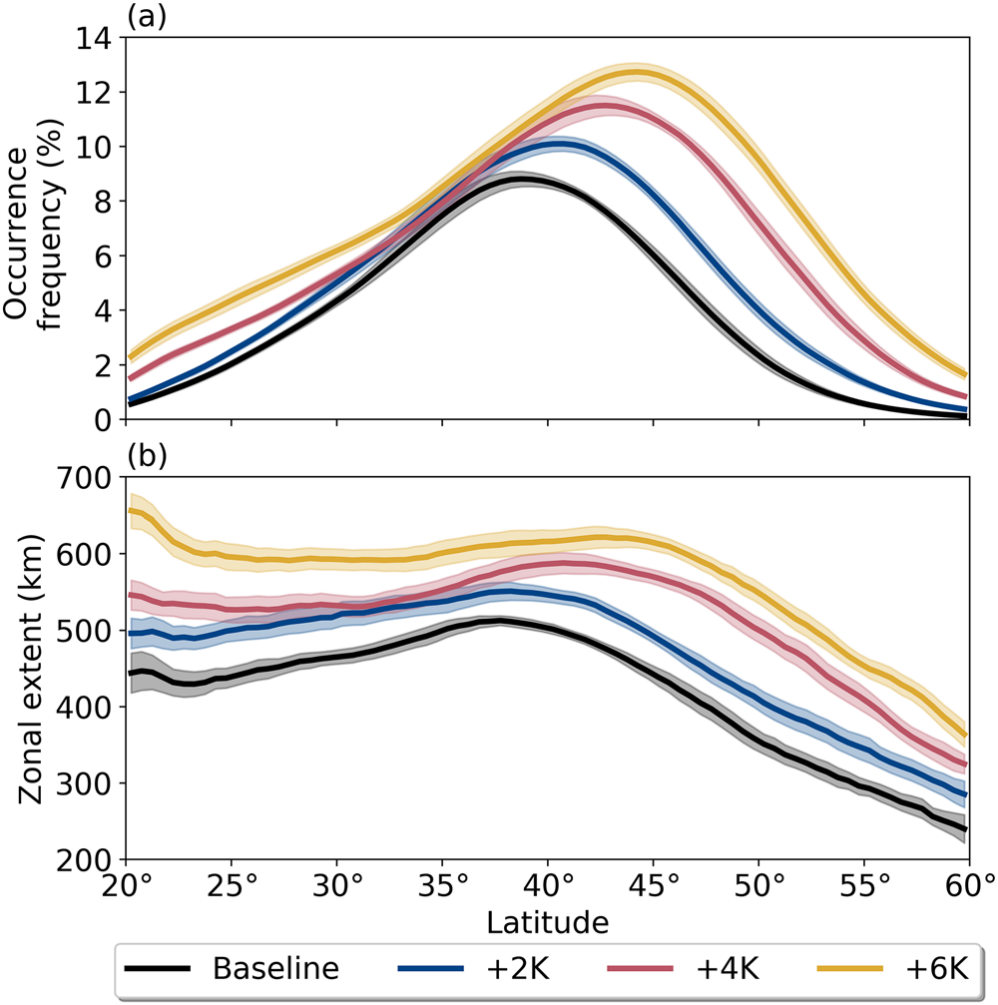
Zonal mean AR (a) occurrence frequency and (b) zonal extent for each SST run. Shading shows the 95% confidence intervals, computed with respect to N=20 three‐month ensembles.

Figure 1 shows a snapshot of TE‐identified ARs against fields of (a) IVT and (b) the Laplacian of IVT for reference. Note that IVT values within AR contours always meet or exceed the typical AR criteria of 250 kg/m/s, even without this threshold being explicitly enforced (Figure 1a). To capture the bulk AR climatology for each run, we also compute an AR occurrence frequency (OF), defined here as the percent of time steps in which TE has identified AR conditions at any given latitude‐longitude grid point. Since these results are independent of zonal coordinate, we show the zonal mean AR OF in Figure 2a. With each 2 K increase in SST, AR OF both increases overall and experiences a robust poleward shift in its maximum. This shift is likely driven by a similar poleward shift in the location of the eddy‐driven jet, which we discuss in section 3.1. Meanwhile, the overall enhancement in AR OF under warming SST conditions can be due to changes in the number of ARs, the zonal extent of AR objects, and/or an increase in the average duration of ARs. We find that the largest contribution comes from the average zonal extent of individual AR objects (i.e., the average number of grid points in the zonal direction occupied by an individual AR object; nominally a measure of AR width without taking AR axis into account), which expands systematically as SSTs warm, especially in the lowest and highest latitudes of the test domain (Figure 2b). Espinoza et al. (2018) similarly reported enhanced AR OF due to expanding AR length and width under climate change conditions using an independently developed ARDT.

The extent of the AR “widening” produced by TE can be explained in part by multiplicative IVT increases since the multiplicative factor carries over into the Laplacian, but it is unlikely to be the only driver: Assuming that AR IVT exhibits a Gaussian profile, it can be shown that a uniform 50% increase in IVT only results in a ∼1% wider AR (Text S1). In any case, the increased width of AR objects does imply some care needs to be taken when assessing zonal mean AR statistics—namely, the added points at the periphery of the AR objects are likely to be different in character than the AR core, which is characterized by the most extreme values of IVT. Subsequently, our analysis examines both the zonal mean statistics and histograms of relevant fields.

### 2.3 Filtering Out Tropical Cyclones

One pitfall associated with the TE AR detection criteria used here is the identification as ARs of bands of high IVT associated with TCs. While ARs can occur in association with TCs and extratropical transition (e.g., Sodemann & Stohl, 2013), our analysis focuses on AR conditions alone and not on related phenomena. Restricting AR detection poleward of 20° removes a substantial proportion of TCs and TC‐like objects, but in order to ensure these objects are not incorporated in our analysis we additionally use a separate detection algorithm to filter them (Ullrich & Zarzycki, 2017; Zarzycki & Ullrich, 2017). TE's TC detection is used to mask out all variables within an eight‐degree radius of grid points which meet the following criteria:

1. They are the most intense local minimum of sea‐level pressure (SLP) within a 2.0° great‐circle distance.
2. Their SLP increases by at least 375 Pa over a 3.6° radius.
3. Their 300 minus 500 hPa geopotential height thickness decreases by six meters over 7.5°. This criterion acts so as to only detect warm core storms and not eliminate extratropical cyclones.

Figure 1 shows an example TC detected by TE against fields of (a) IVT and (b) the Laplacian of IVT for reference. For further details on how TE detects TCs, we refer readers to Ullrich and Zarzycki (2017) and Zarzycki and Ullrich (2017). We note here that while removing TCs did not change our overall results substantially, it did remove spuriously high values of IVT, low‐level wind speeds, and precipitation from AR zonal mean statistics between 20° and 25°.

### 2.4 Data and Statistical Tests

Due to zonal uniformity and symmetry of SSTs across the equator, AQPs lend themselves nicely to meridional distributions of zonal mean statistics. Hence many statistics are presented in this form and due to the aforementioned hemispheric symmetry, are shown only for the northern hemisphere (although southern hemisphere statistics are incorporated in these plots as well). Furthermore, since we do not track ARs in time, all analysis on AR variables (e.g., AR precipitation) is performed on a grid point basis, rather than over the entire AR object throughout all or part of its lifecycle. Therefore, all meridional distributions of AR variables discussed here may be better characterized as distributions of variables under “AR conditions” (i.e., TE has identified that grid point as belonging to an AR) in which all non‐AR grid points are excluded from analysis. We evaluate significance throughout this manuscript at the 95% confidence level with respect to 3‐monthly ensemble members with the northern and southern hemispheres treated as independent samples ( N=20). This is analogous to a series of seasonal‐length ensembles.

Since some variables exhibit strong meridional variations in terms of their relative response, we stratify our analysis into four distinct, approximately equal‐area latitude bands: Lower subtropics (LST; 20.25–26.75°), upper subtropics (UST; 30.25–37.75°), lower midlatitudes (LML; 38.25–46.75°), and upper midlatitudes (UML; 47.25–57.25°). The relatively large gap between LST and UST exists because of a complex precipitation response in the area between those bands, as seen in Figure  10; we reserve discussion of precipitation for section 3.5.

## 3 Results and Discussion

We begin our discussion in section 3.1 with a brief summary on the midlatitude circulation for each SST run, as it is important for understanding the AR environment. Next, section 3.2 describes findings on IVT, the variable on which AR detection is conditioned and one of the most prominent features of ARs. We follow this up by roughly considering IVT as the product of IWV and low‐level wind, thus allowing us to separate IVT into its thermodynamical (IWV) and dynamical (wind) contributions. These contributions are then analyzed in detail in sections 3.3 and 3.4, respectively. Finally, AR precipitation is analyzed in section 3.5.

### 3.1 Circulation Response

The midlatitude circulation provides the large‐scale dynamical background for ARs. We characterize the midlatitude circulation in terms of three metrics: the Hadley cell (HC) edge, the subtropical jet (STJ), and the eddy‐driven jet (EDJ). The purpose of this section is to provide a broad overview of the major circulation responses to SST increases in order to provide context for the AR responses described in later sections.

**Figure 3:**
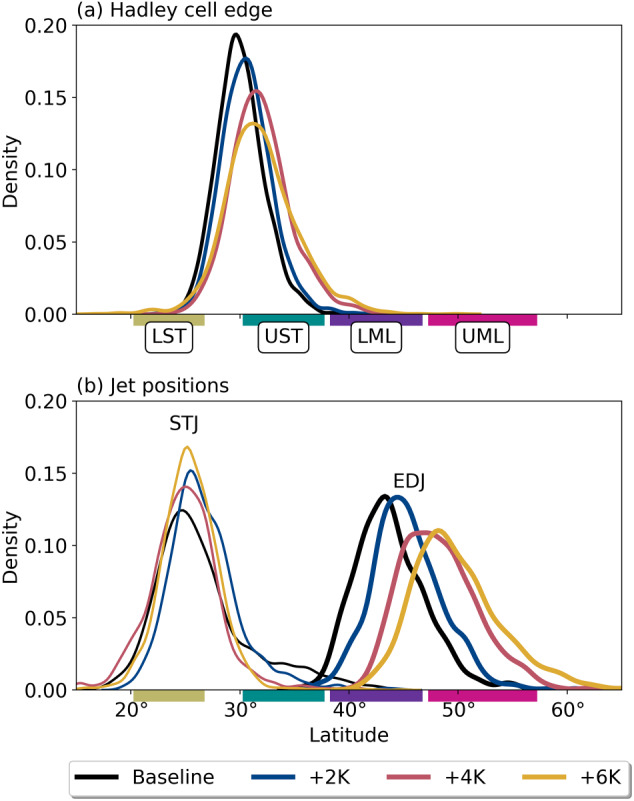
Kernel density estimates (KDEs) of (a) Hadley cell edge for each run and (b) subtropical jet (STJ; thin lines) and eddy‐driven jet (EDJ; thick lines) positions. Colored boxes and accompanying labels on the *x*‐axis denote the analysis subregions described in section 2.4, and are shown here for reference.

The HC edge defines the poleward extent of the region of subsidence associated with the HC's descending branch, where strong static stability suppresses precipitation. To locate it, we follow the work of Davis and Birner (2016) who define it as the latitude at which the column‐integrated zonal‐mean meridional stream function (MMS) first disappears poleward of the deep tropical MMS extrema. We use bilinear interpolation to find the precise latitude of the HC edge when it is between grid points, as in Davis and Birner (2016). Figure 3a depicts a kernel density estimate (KDE) of the daily‐mean HC edge. Despite a slight discontinuity between the +4 K and +6 K runs, the HC edge generally shifts poleward as SST warms, a result which has been observed and analyzed previously (Frierson et al., 2007; Lu et al., 2007; Maher et al., 2020; Shaw & Voigt, 2016; Tandon et al., 2013; Vallis et al., 2015).

Whereas the HC edge broadly defines regions of convergence and divergence through the tropics and subtropics, the tropospheric jets act as dynamical guides against which cyclones, and the closely related ARs, form and propagate. Statistics related to AR occurrence, landfall location, and moisture transport are impacted by the prevailing jet regime for a given ocean basin (Kim et al., 2019; Payne & Magnusdottir, 2015; Shields & Kiehl, 2016). Thus, we provide a short summary of EDJ and STJ statistics here, beginning with the EDJ.

To locate the EDJ, we use the method described in Woollings et al. (2010): We (1) perform vertical averaging from 925 to 700 hPa of the daily mean zonal wind fields between 15° and 70° and (2) smooth the zonal mean using a 10‐day Lanczos filter with a 61‐day window (Duchon, 1979). We then denote the latitude of the daily maximum of this field as the EDJ position. Accumulated over the complete simulation, the KDE of the EDJ position is given in Figure 3b. We find that the EDJ position shifts poleward as SST increases, an unsurprising result given the uniform SST increases and the strengthening upper‐level temperature gradient shown in Figure 7 (e.g., Shaw & Voigt, 2015). As the EDJ shifts poleward, it enhances static stability through the subtropics, similarly pushing storm tracks and the HC edge poleward (Butler et al., 2010; Chang et al., 2012; Yin, 2005). Hence, the shift seen here in the EDJ is consistent with our results for the HC edge and AR occurrence frequency, both of which shift poleward with respect to the Baseline run (Figure 5).

Last, we find the STJ as in Davis and Birner (2016), who define it as the most equatorward zonal mean zonal wind maximum below 50 hPa after subtracting the surface zonal wind component to distinguish it from the EDJ. We remove the 850 hPa wind from the column instead as in Maher et al. (2020) and similarly find a distinct STJ core (Figure S4). Figure 3b shows KDEs of daily STJ position for each SST run. Unlike the HC edge's systematic poleward shift as SST increases, the STJ location shows a discontinuous response characterized by a relatively large poleward movement in the +2 K scenario but smaller poleward movements in the +4 K and +6 K scenarios, when measured with respect to the Baseline run. Even though the HC edge and STJ are frequently co‐located, a “de‐coupling” characterized by differing meridional shifts in the position of each has been described before (Davis & Birner, 2013; Maher et al., 2020). We also cannot rule out difficulties with separating the STJ from the EDJ (Medeiros et al., 2016), although this is less of an issue in the +4 K and +6 K experiments (Figure 3b). Another systematic response to uniformly warming SSTs is a strengthening of the STJ, as evidenced by higher zonal mean zonal winds in its core (Figure S4). The STJ strengthening is likely related to the enhanced upper‐level meridional temperature gradient in the subtropics as SSTs warm (Figure 7.)

### 3.2 Vapor Transport

**Figure 4:**
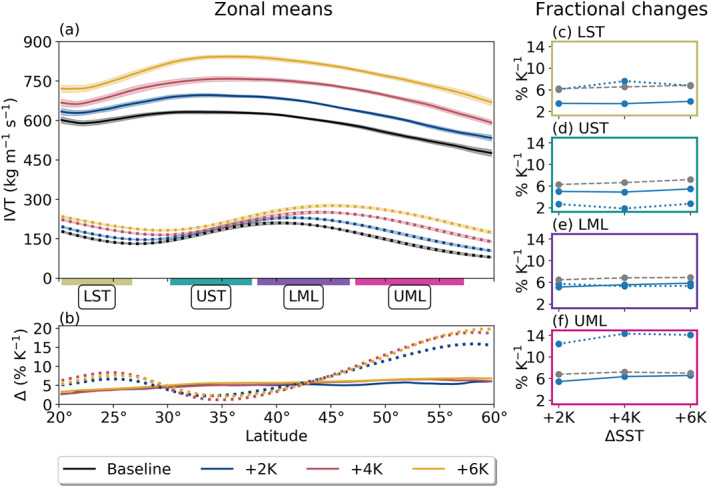
(a) Meridional distributions of zonal mean AR (solid) and non‐AR (dotted) IVT. Shading shows 95% confidence intervals. (b) Relative differences with respect to the baseline SST (% K^−1^), using the same line color and style conventions. (c–f) Area‐weighted mean relative change per K SST increase (blue; line style conventions as before). Gray dashed lines show changes in near‐surface *e*^∗^ as predicted by the CC relation computed with respect to the prescribed uniform SST increases, which we show here for reference.

Figure 4 shows (a) meridional distributions of zonal mean IVT for all runs, as well as (b) the relative change between each warming run and the Baseline (expressed as % per K). Solid lines show zonal averages over grid points in which AR conditions are present, whereas the dotted lines show zonal averages over non‐AR grid points only. Finally, (c)–(f) show area‐weighted fractional changes for each analysis subregion described in section 2.4. We find that both AR and non‐AR IVT increases with SST, though the response is not entirely uniform. While the change in AR IVT shown in Figure 4b appears virtually flat and typically increases quite linearly at approximately 5% K^−1^, the change through the LST is only about half as strong. The relatively uniform increase across latitudes in AR IVT contrasts the change in non‐AR IVT, which is characterized by both an overall increase as well as poleward shifting maxima (moving from 40° to 46° under the +6 K scenario) concordant with shifts in the EDJ position (section 3.1). In terms of overall fractional changes, it is notable that non‐AR IVT increases at higher rates than AR IVT in the LST and UML subdomains, at lower rates in the UST, and at similar rates in the LML.

**Figure 5:**
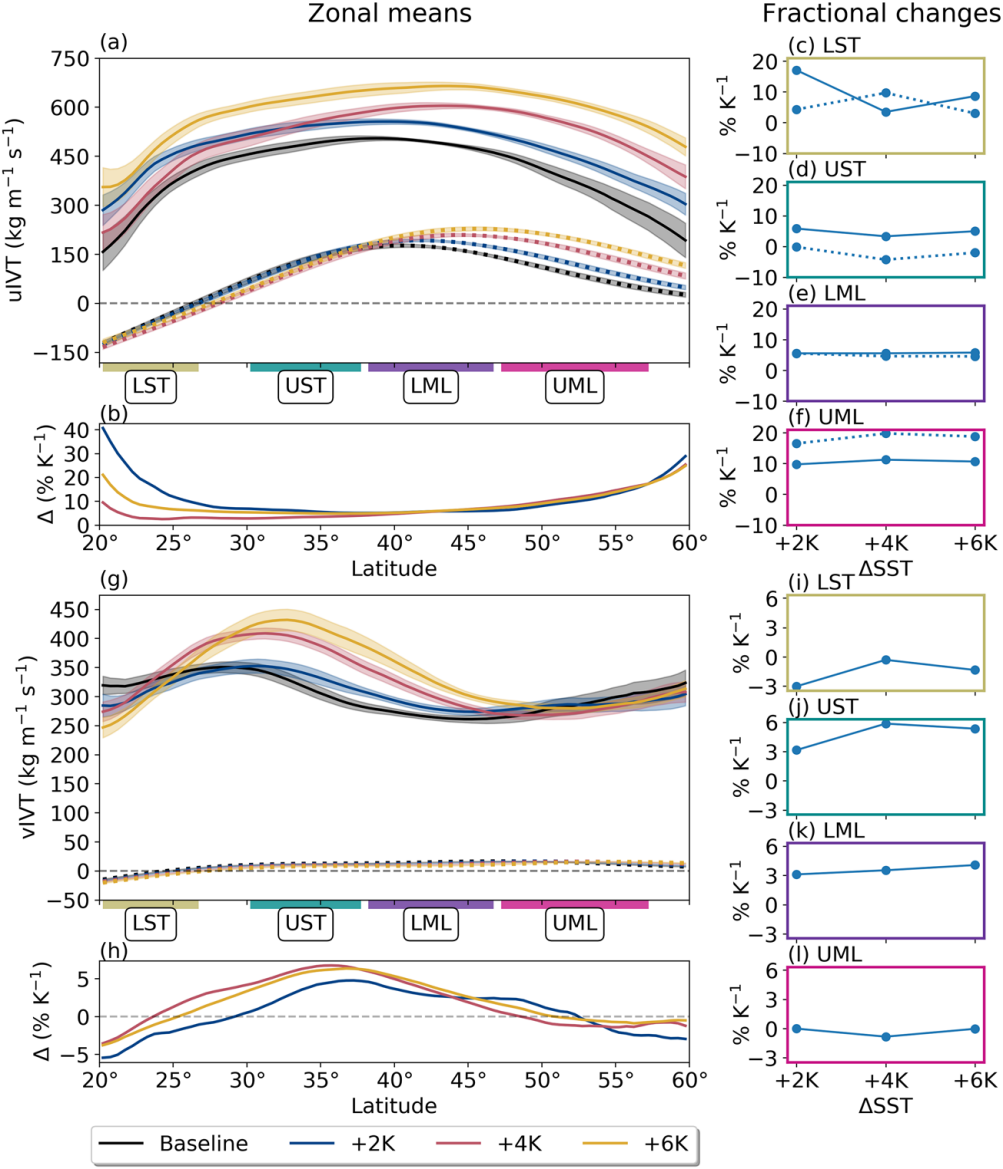
As in Figure 4, but for (a–f) zonal IVT (uIVT) and (g–l) meridional IVT (vIVT) components. Note the differences in ordinate scales. Also note numerical issues which prevented us from plotting some non‐AR quantities: (1) Non‐AR uIVT is consistently near‐zero between the LST and UST, resulting in an artificial inflation of relative changes in subplot (b), though the subregion means (c–f) were possible; (2) non‐AR vIVT has a similar problem, though the values pass through the y‐intercept in the LST; hence, we could plot neither the zonal (h) nor the regional (i–l) fractional changes.

We investigate changes in zonal mean IVT first by breaking it up into its zonal (uIVT) and meridional (vIVT) components (Figure 5). Perhaps the most obvious feature emerging from this decomposition is the somewhat complementary relative changes between AR uIVT and vIVT: The largest relative increases in uIVT occur at the LST and UML, where vIVT decreases slightly; likewise, the largest increases in vIVT occur through the UST and LML subregions, where uIVT shows the smallest fractional change. Specifically, when it comes to changes in the direction of vapor transport, we show here that increasing SST leads to increasingly westerly AR IVT through the LST and UML but does not substantially change transport direction through the UST and LML, where both uIVT and vIVT experience similar fractional increases. Nonetheless, the relatively localized increase in vIVT in the UST suggests an intensification in AR vapor advection from the subtropical moisture reservoir as SSTs increase. The changing pattern of AR vIVT out of the LST tracks with observed shifts in the STJ for each SST scenario (Figure 3b)—namely, AR vIVT is enhanced on the cyclonic side of the jet and suppressed on the anticyclonic side—suggesting that the strengthening zonal flow of the STJ reduces AR‐modulated interactions between the equatorward and poleward sides of the jet.

In non‐AR regions, the poleward shift in IVT is exclusively due to an increase and poleward shift in westerly transport (uIVT), as meridional transport remains small (or even shows a small statistically insignificant decrease outside of the LST), a change likely facilitated by the poleward shift in the EDJ (Figure 3). Given that vIVT in non‐AR regions is small, moisture transport into the midlatitudes appears increasingly dominated by ARs as SSTs increase. Since no corresponding increase in moisture flux occurs in the UML, we thus expect that precipitation increases in the LML will be largely due to increases in AR precipitation.

### 3.3 Thermodynamic Response

**Figure 6:**
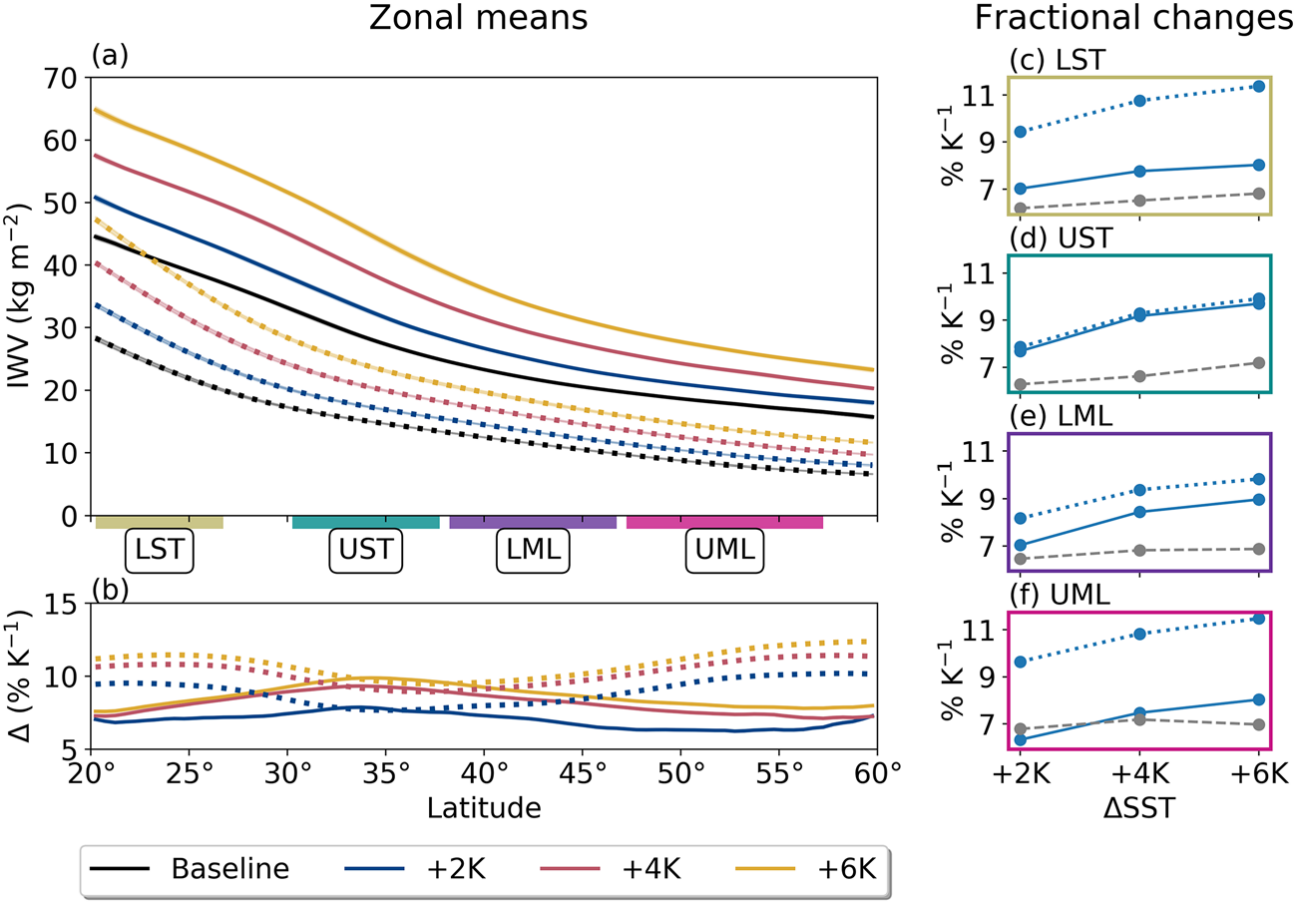
As in Figure 4, but for IWV.

Figure 6 shows zonal mean IWV and its fractional change under uniformly increased SST forcing. AR and non‐AR IWV both increase systematically with warming SSTs, with non‐AR IWV changing at higher fractional rates than AR IWV in all domains except the LML, in which AR and total IWV change approximately equivalently. As with the CC relation for near‐surface *e*^∗^ (gray lines on Figures 6c–6f), IWV increases are enhanced slightly for larger SST increases. However, the magnitude of IWV enhancement exceeds CC predictions for near‐surface *e*^∗^ when conditioned on SSTs (except for in the +2 K UML).

**Figure 7:**
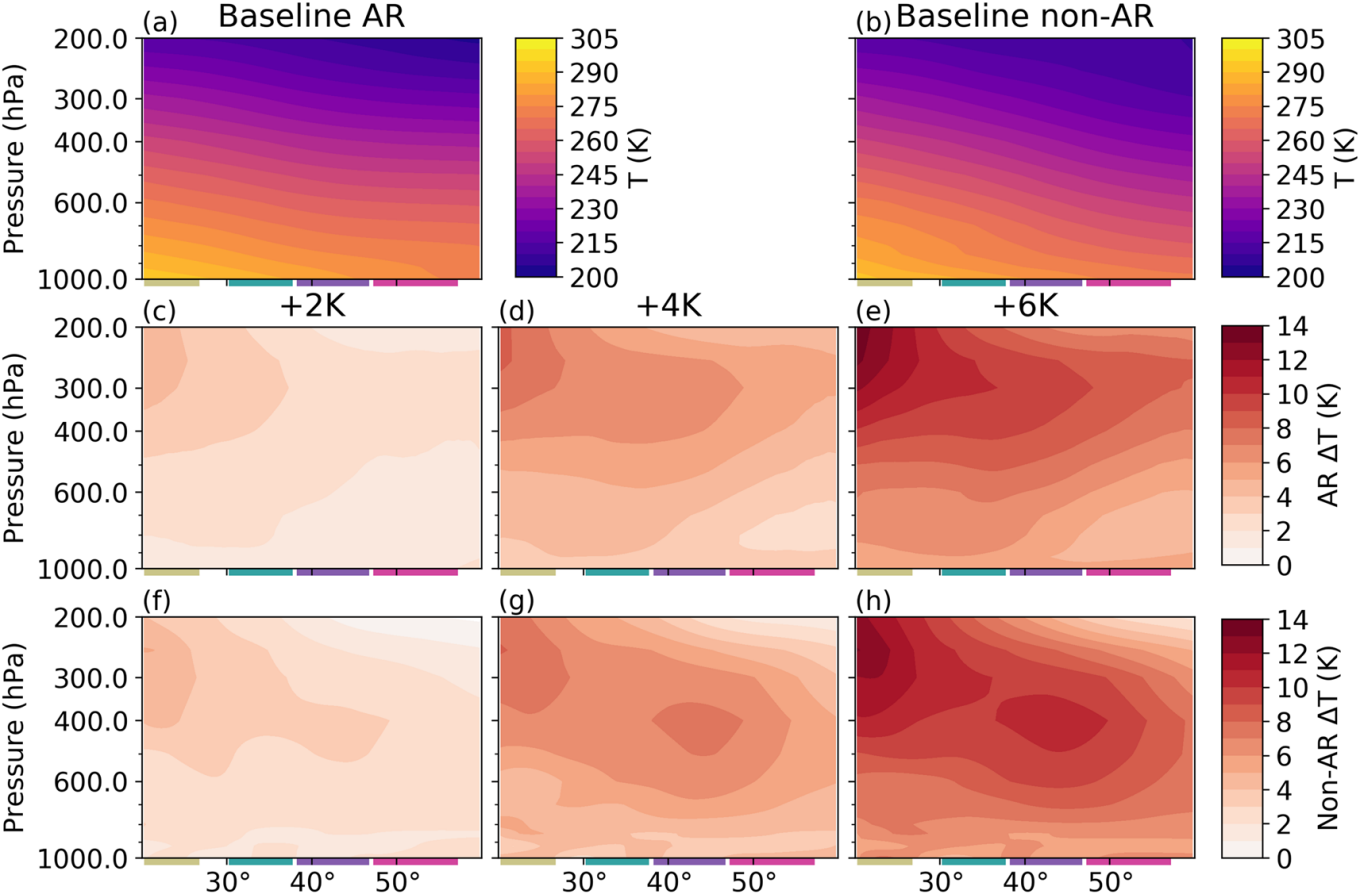
Vertical distributions of zonal mean absolute temperature in (a) AR and (b) non‐AR grid points. (c–e) Vertical distributions of the absolute change in absolute temperature in AR grid points. (f–h) Vertical distributions of the absolute change in absolute temperature in non‐AR grid points. Boxes delineating subregions are as for the previous figures, and are shown for reference.

Similar “super‐CC” moistening rates in ARs have been observed before (Gao et al., 2015). However, the idealized aquaplanet used in this paper allows us to more easily elucidate the relevant physical linkages. We begin by examining column temperatures: Figure 7 shows vertical profiles of zonal mean absolute temperature changes in AR and non‐AR grid points. Although these profiles incorporate not only the cores of ARs but also their peripheries, the vertical distributions here nonetheless allow us to unravel one of the physical drivers of the zonal mean IWV signals seen in Figure 6. We find that the temperature response for AR grid points in all subregions and non‐AR grid points through the LST and UST is consistent with a damping of the moist‐adiabatic lapse rate, which can be shown to occur under surface warming conditions as a result of enhanced latent heat release aloft (Payne et al., 2020; Siler & Roe, 2014). Meanwhile, the midtropospheric warming maximum in non‐AR grid points in the LML and UML is similar to that seen in previous studies citing more complicated physical mechanisms for tropospheric warming in the midlatitudes which are beyond the scope of this manuscript (e.g., O'Gorman & Singh, 2013).

**Figure 8:**
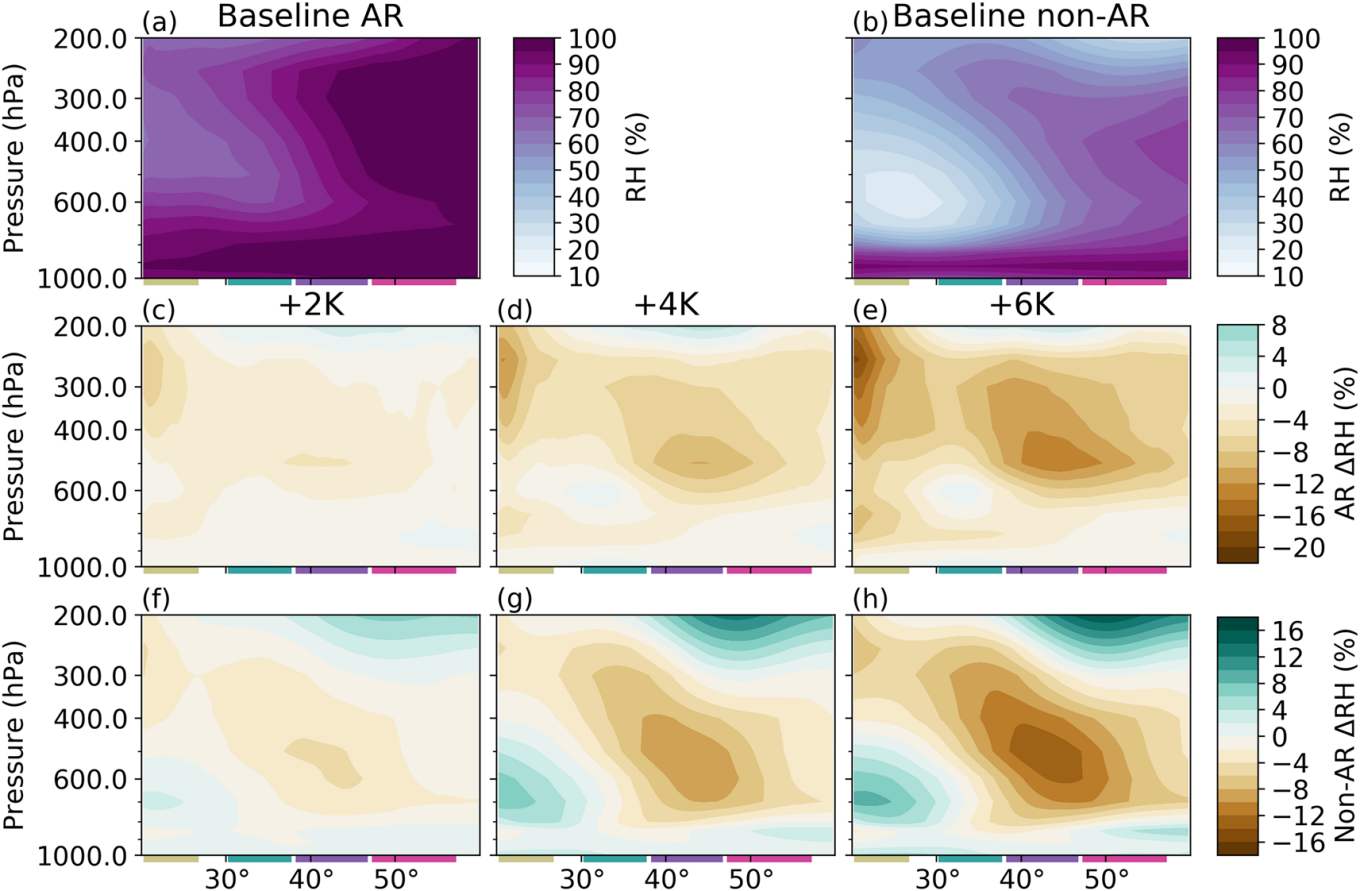
Same as Figure 7, but for relative humidity (RH; %).

While the temperature analysis facilitated by Figure 7 provides a qualitative evaluation of model output with respect to theoretical predictions, it does not address why non‐AR IWV tends to increase at higher rates than AR IWV (Figure 6). To investigate this, we leverage relative humidity (RH), an ideal variable in this scenario since it directly shows the departure of specific humidity from saturation specific humidity under a given SST increase, providing important context for the differing responses between AR and non‐AR IWV. To that end, Figure 8 shows vertical profiles of the absolute change of RH in AR and non‐AR grid points for each SST run. These profiles show that RH decreases through most of the column and at most latitudes whether or not we select for AR conditions, though the decrease is generally larger at AR grid points. Since oversea midtropospheric RH decreases through the subtropics and midlatitudes have been noted before (e.g., O'Gorman, 2015), we focus our discussion here on changes within ARs. For a general RH decrease in AR grid points through the LST and UST, a role is likely played by largely sub‐CC increases in surface evaporative fluxes (Figure S6), which we note are consistent with previous thermodynamic studies (e.g., Held & Soden, 2006; O'Gorman & Muller, 2010; O'Gorman et al., 2012). Compounding this effect is an overall decrease in column moisture flux convergence (MFC) into AR grid points in the subtropics which is particularly strong in the LST (Figure S7) and likely arises as a result of the poleward expansion of the HC edge (Figure 3). Meanwhile, despite decreasing condensation of AR moisture onto the surface in the LML and UML (Figure S6), the sub‐CC increases in MFC through these regions (Figure S7) also likely contribute to RH decreases here. The only location where ARs get closer to saturation under SST increases is the UST midtroposphere, though the RH increase here is very slight and is likely facilitated by enhanced meridional vapor advection out of the LST (Figure 5h).

### 3.4 Dynamic Response

**Figure 9:**
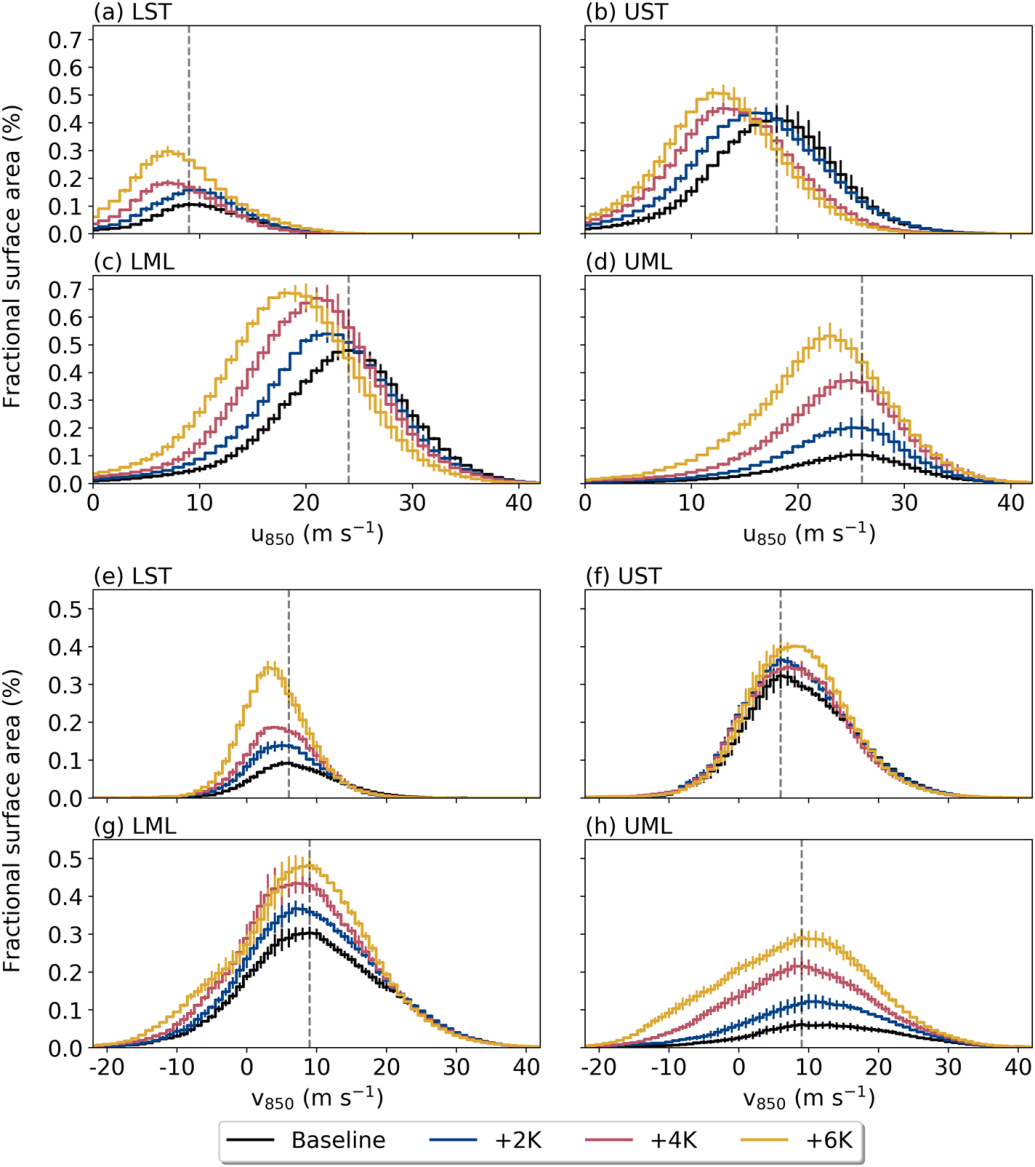
Histograms of AR (a–d) zonal wind at 850 hPa (u_850_) and (e–h) meridional wind at 850 hPa (v_850_) in each analysis subregion, with spacing at 1 m/s; *y*‐axis shows the fractional area of the subregion occupied by a particular bin value. Steps show the median of the 3‐month ensemble members, while error bars show the inter‐quartile range with respect to N=20 ensemble members. The gray, dashed lines show the mode of the baseline histogram for reference.

To broadly understand the dynamical component of AR IVT, we examine AR winds at the 850 hPa level. Zonal means show a robust decrease in AR 850 hPa wind speeds (Figure S5), which we decompose into histograms of zonal and meridional winds taken over all AR points within each subregion (Figure 9). These distributions reveal that zonal winds at AR points generally weaken as SST increases, with the most robust of these decreases occurring through the UST and LML. Since the EDJ shifts from primarily occurring in the LML to the UML under higher SST conditions (Figure 3b), this slowing is likely related to a poleward shift in the strongest steering winds. Meanwhile, 850 hPa meridional winds in ARs show a more complicated response, characterized less by a systematic shift toward lower values and more by an increased sampling of grid points featuring weak or even equatorward meridional winds. That is, the upper tails of all four histograms (>20 m/s)—representing points largely drawn from the AR core—exhibit essentially no change in meridional wind speed.

As stated earlier, we roughly consider IVT to be the product of IWV and low‐level wind speed. With this in mind, the decreased magnitude of zonal mean IVT increases compared to IWV increases suggests that slowing winds attenuate the IVT response. We compactly show the compensatory relationship between IWV and low‐level winds on IVT by returning to the weighted area‐mean fractional changes of IVT and IWV (Figures 4 and 6) for each of our analysis subregions and compute the same for 850 hPa wind speed (*U*_850_). Following this, we describe a relative change in IVT as

(2) $$\frac{\Delta IVT}{IVT}=\frac{\Delta IWV}{IWV}+\frac{\Delta U_{850}}{U_{850}}+r$$

where *r* is the residual value. We plug in the area‐weighted fractional changes for IVT, IWV, and *U*_850_ to Equation 2, the results of which are summarized in Table 1.

**Table 1:** Area‐Weighted Mean Relative Changes for IVT, IWV, and *U*_850_ for Each Latitude Band in All SST Experiments

		$\frac{\Delta IVT}{IVT}$	$\frac{\Delta IWV}{IWV}$	$\frac{\Delta U_{850}}{U_{850}}$	*r*
LST	+2 K	**3.49** (6.04)	**7.03** (9.44)	− **5.51** (−2.11)	**1.97** (−1.29)
	+4 K	**3.44** (7.59)	**7.77** (10.76)	− **4.33** (−1.07)	**0.00** (−2.10)
	+6 K	**3.87** (6.76)	**8.03** (11.37)	− **4.22** (−1.69)	**0.06** (−2.92)
UST	+2 K	**4.98** (2.68)	**7.69** (7.86)	− **2.96** (−4.10)	**0.25** (1.08)
	+4 K	**4.87** (1.86)	**9.18** (9.29)	− **3.53** (−4.98)	− **0.78** (−2.95)
	+6 K	**5.44** (2.74)	**9.70** (9.91)	− **3.12** (−4.13)	− **1.14** (−3.04)
LML	+2 K	**5.11** (5.71)	**7.03** (8.17)	− **2.14** (−1.43)	**0.22** (−1.03)
	+4 K	**5.53** (5.26)	**8.44** (9.38)	− **2.76** (−2.47)	− **0.15** (−1.65)
	+6 K	**5.82** (5.32)	**8.97** (9.84)	− **2.50** (−2.37)	− **0.65** (−2.15)
UML	+2 K	**5.43** (12.39)	**6.33** (9.65)	− **0.78** (3.02)	− **0.12** (−0.28)
	+4 K	**6.35** (14.28)	**7.47** (10.83)	− **1.15** (3.10)	**0.03** (0.35)
	+6 K	**6.55** (14.04)	**8.03** (11.48)	− **1.23** (2.26)	− **0.25** (0.30)

*Note.* We also show the residual value, *r*, as expressed in Equation 2. AR values are expressed in boldface, while non‐AR values are contained in parentheses. All values have units of % K^−1^ conditioned on the given uniform SST increase.

Performing this simplified analysis allows us to see the relative roles of thermodynamics (i.e., moisture fields) and dynamics (in this case, wind magnitude) in modulating AR and large‐scale vapor transport. In particular, it allows us to see the opposition between the thermodynamic and dynamic components under warmer SST regimes. The generally small magnitudes of the *r* values in AR grid points indicates that absent changes in wind speed, AR IVT increases are approximated by those in AR IWV, a result which agrees well with several previous analyses (Dettinger, 2011; Gao et al., 2015, 2016; Lavers et al., 2013; Payne et al., 2020; Warner et al., 2014). Non‐AR *U*_850_ values illustrate this especially well, as their changes do not represent a systematic decrease so much as a poleward‐shifting maximum. As a result, non‐AR IVT values in the LST, UST, and LML regions all increase at rates lower than non‐AR IWV, while increases in UML non‐AR IVT far exceed those of non‐AR IWV, due to enhanced *U*_850_ at these high latitudes.

### 3.5 Precipitation

We move on now to perhaps the most widely associated feature of ARs. We begin by examining zonal means within AR and non‐AR grid points, as in previous sections. From here, we examine the drivers of precipitation and its changes first by applying a simplified physical model of precipitation analogous to that found in Table 1. We follow this up by examining distributions of AR precipitation and vertical velocities in more detail. Last, we characterize changes in AR precipitation independent of changes in AR areal extent.

#### 3.5.1 Bulk Properties and Drivers of AR Precipitation

**Figure 10:**
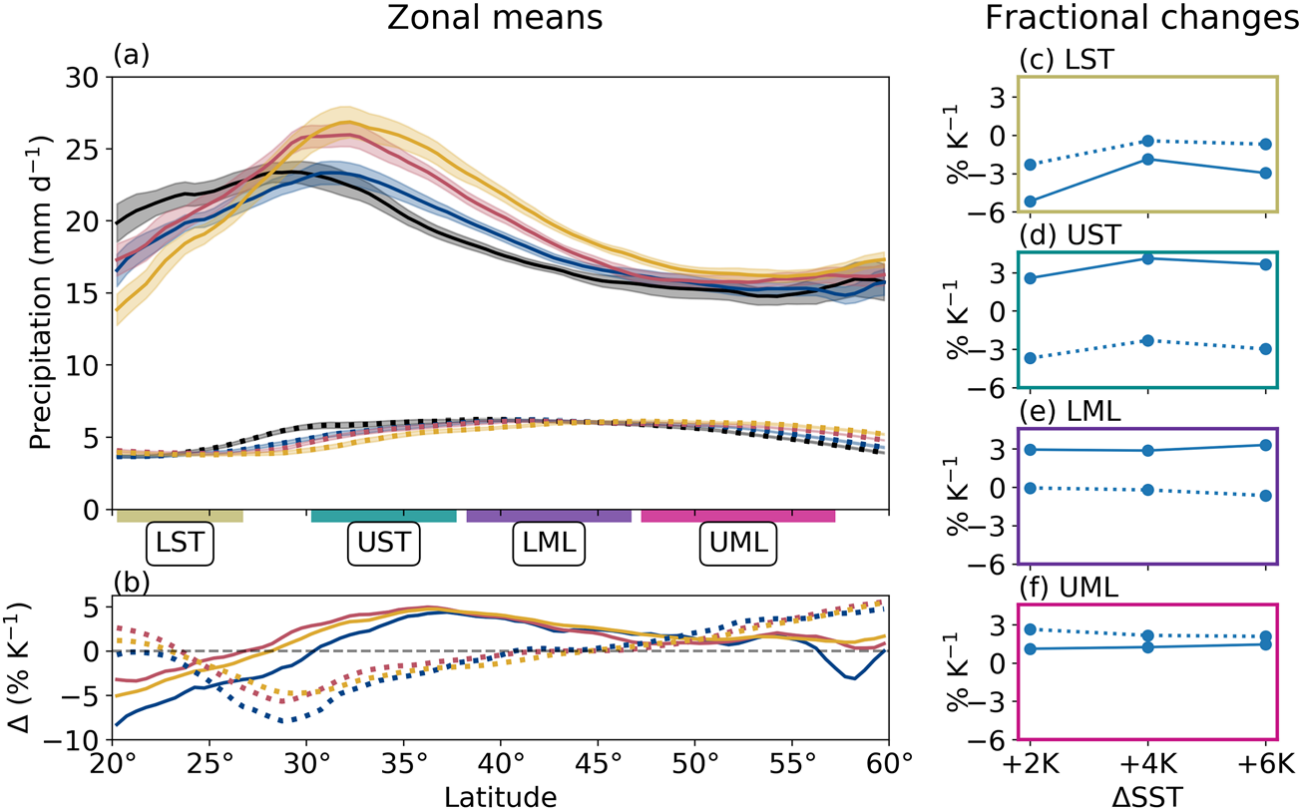
The same as Figure 4, but for 3‐hourly average precipitation rate.

Figure 10 shows zonal mean 3‐hourly average precipitation rates as well as the fractional change of those rates with respect to the prescribed SST increase. Non‐AR precipitation exhibits a pattern consistent with the poleward shifts in the HC edge and the EDJ position (Figure 3), which together serve to enhance moisture flux convergence (MFC) in the UML and suppress it through the UST, though the MFC increase in the UML is very slight (Figure S7). Generally speaking, this pattern is consistent with other thermodynamic scaling studies of mean precipitation rates (e.g., Held & Soden, 2006; O'Gorman & Schneider, 2009b), so we turn our focus now to AR precipitation, whose response shows a similar pattern to non‐AR precipitation but with marked meridional shifts. For instance, like fractional changes in AR vIVT (Figure 5h) the spatial pattern of the AR precipitation response through the LST and UST (Figure 10b) tracks more closely with observed shifts in the STJ position than in the HC edge (Figure 3). Hence, while decreasing AR precipitation through the LST may be related to the expansion of the HC's descending branch through this subregion, we also suspect effects associated with changing patterns of convergence and divergence in the vicinity of the STJ.

To contextualize our findings on AR precipitation, we perform a simplified analysis similar to that performed for IVT in section 3.4. To do so, we assume the local precipitation rate (*P*) of AR grid points over the ocean is roughly equal to the vertically integrated condensation rate; we can thus characterize it with the following proportionality:

(3) $$P∼\int_{0}^{p_{s}}\omega {\left.\frac{dq^{*}}{dp}\right|}_{\theta_{e}}dp$$

where *ω* is the vertical velocity in pressure coordinates and *dq*^∗^/*dp* is the vertical gradient of specific humidity evaluated along a moist adiabat with constant equivalent potential temperature *θ*_*e*_. Given the approximately moist‐neutral thermal stratification of strong ARs (Ralph et al., 2017), *dq*^∗^/*dp* can be integrated in pressure coordinates to yield the surface specific humidity, $q_{sfc}^{*}$; since ARs are approximately saturated near the surface (Figure 8), we set $q_{sfc}^{*}=q_{sfc}$ at AR grid points. We furthermore simplify this relation by evaluating *ω* only at the 700 hPa level. Accordingly, a fractional change in AR precipitation can be expressed as

(4) $$\frac{\Delta P}{P}=\frac{\Delta q_{sfc}}{q_{sfc}}+\frac{\Delta \omega_{700}}{\omega_{700}}+r$$

As with the earlier IVT analysis (Table 1), we compute terms with respect to weighted areal means within each analysis subregion. The results of this analysis are summarized in Table 2. Generally speaking, residual values tend to be small (Table 2); in addition to other assumptions which went into Equation 4, we recognize the finding here that AR grid points are not exactly moist‐neutral (section 3.3) likely contributes to the residuals. Regardless, Equation 4 captures changes in zonal mean AR precipitation rates reasonably well.

**Table 2:** As for Table 1, but for Equation 4 Instead and Only Considering AR Grid Points

		$\frac{\Delta P}{P}$	$\frac{\Delta q_{sfc}}{q_{sfc}}$	$\frac{\Delta \omega_{700}}{\omega_{700}}$	*r*
LST	+2 K	−5.17	5.67	−11.89	1.05
	+4 K	−1.87	6.31	−7.41	−0.77
	+6 K	−2.96	6.63	−7.87	−1.72
UST	+2 K	2.57	6.59	−3.38	−0.64
	+4 K	4.12	7.17	−3.13	0.07
	+6 K	3.66	7.58	−3.25	−0.68
LML	+2 K	2.94	6.47	−4.08	0.56
	+4 K	2.87	6.93	−5.25	1.19
	+6 K	3.31	7.40	−4.31	0.22
UML	+2 K	1.11	6.66	−4.97	−0.57
	+4 K	1.24	7.09	−5.35	−0.50
	+6 K	1.45	7.56	−4.84	−1.27

*Note.* A negative change in the *ω*_700_ ratio means less vigorous upward motion (i.e., vertical velocities are getting more positive). Additionally, only AR values are shown because non‐AR *ω*_700_ approaches 0 at some points in the UST, where relative changes will approach infinity (and thereby artificially inflate area‐mean values).

We find the most substantial weakening in *ω*_700_ in the LST, coincident with the largest decrease in precipitation rates. Meanwhile, in the UST, where some of the largest precipitation increases occur, we also see the slightest weakening in *ω*_700_ and some of the largest near‐surface *q* increases, suggesting that substantial increases in moisture may help overcome weakening upward motions in some regions. A similar explanation can be used for the LML, where fractional changes in AR precipitation follow a nearly linear increase with SST. While the UML also shows a nearly linear fractional increase in precipitation, the smaller magnitudes overall are facilitated by the stronger suppression of *ω*_700_ here. As for why vertical motions systematically decrease in magnitude under warming SST conditions, this outcome appears to be a consequence of weakening vertical temperature gradients (section 3.3) and a subsequent strengthening in local static stability. We recognize that the enhanced static stability in the UML is likely somewhat inflated by the use of the prescribed SST, since a variety of mechanisms (e.g., atmosphere‐ocean feedbacks, enhanced poleward latent heat flux by ARs and other processes) would normally lead to a larger SST increase here (e.g., Langen et al., 2012; Roe et al., 2015). Regardless, this analysis produces results similar to those for IVT, in that thermodynamic effects alone (*q*_*sfc*_) serve to enhance AR precipitation, while dynamical effects (here *ω*_700_) attenuate this enhancement (and in this case, even compensate for it sufficiently to decrease mean precipitation rates in the LST).

#### 3.5.2 AR Precipitation in More Detail

**Figure 11:**
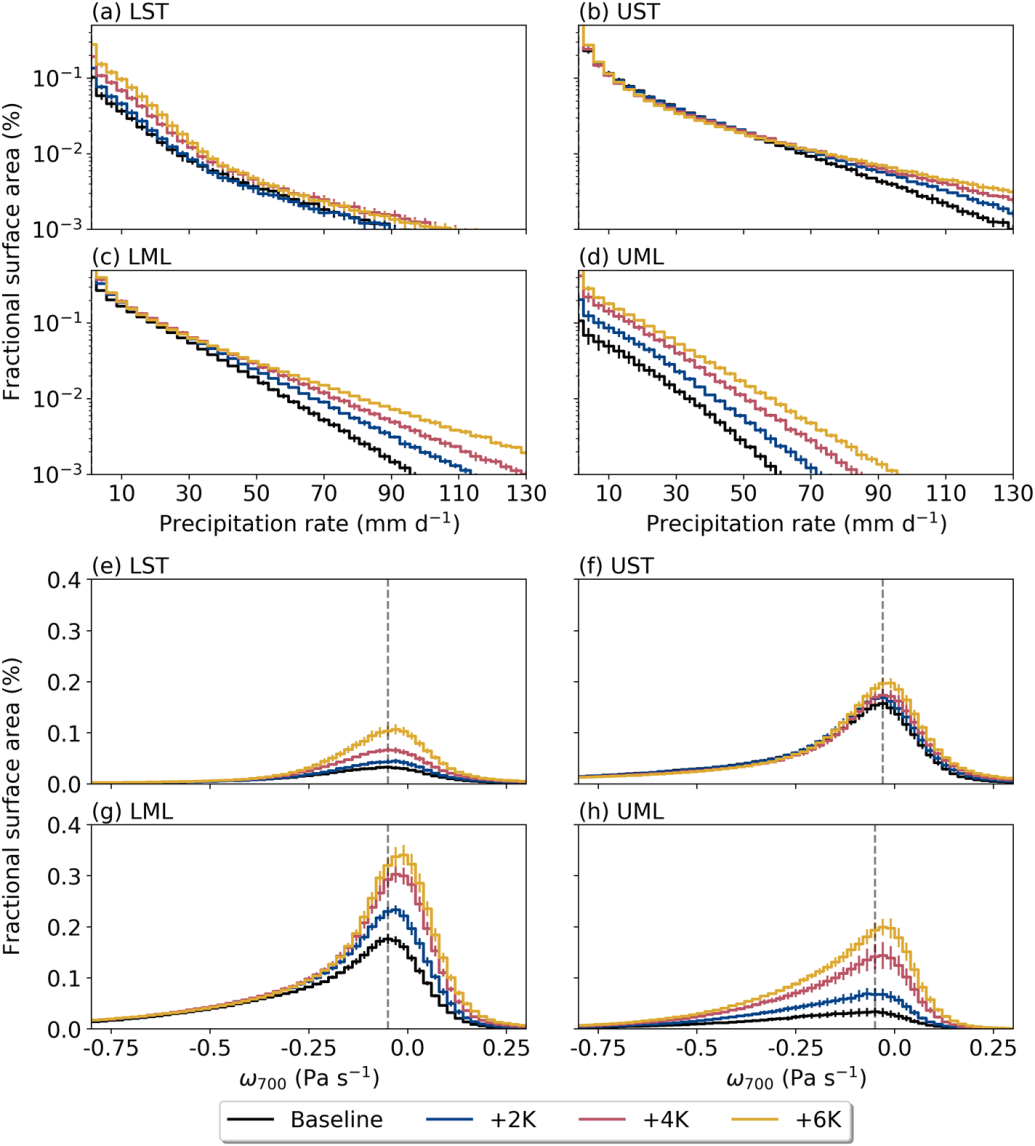
The same as Figure 9, but for 3‐hourly average (a–d) precipitation rates and (e–h) pressure velocities at 700 hPa (*ω*_700_). For (a–d), bin spacing is 1 mm/day with every third bin shown for clarity; we only consider points precipitating with a rate of at least 1 mm/day. For (e–h), bin spacing is 0.01 Pa/s, with every other shown.

We recognize that neither seasonal‐length zonal means of AR precipitation rates (nor large‐scale area integrals of them) are the most informative metric, especially when the most extreme AR events can lead to catastrophic flooding (e.g., Ralph et al., 2006). To that end, Figures 11a–11d show histograms of precipitation rates at AR grid points stratified by analysis subregion, alongside histograms of *ω*_700_ for reference (Figures  11e–11h). We find an overall increase in our sample size for each subregion, consistent with increased AR occurrence and area (Figure 2). In the LST, this increase is notably more significant at precipitation rates below 20 mm/day, accounting for the observed decrease in mean precipitation rates here (Figure 10). Similarly, *ω*_700_ in the LST also exhibits an enhanced sampling of weaker grid points. We attribute this increased sampling of weaker precipitation rates and vertical velocities in this subregion to the large enhancement of AR zonal extent (Figure 2) here: By sampling more grid points at the periphery of AR objects, we also expect to sample grid points exhibiting less vigorous vertical velocities and lower precipitation rates. However, extreme precipitation rates (>70 mm/day) also cover more area within this region as SSTs increase, indicative of an enhancement in extreme precipitation and a larger spread in AR precipitation rates overall.

By contrast, both the UST and LML regions have marked increases in the most intense precipitation rates (>70 mm/day) that are in turn responsible for driving up the mean precipitation rates. Interestingly, we do not see a similar uptick in intense vertical velocities in these subregions; however, as per the analysis presented in Table 1, enhanced AR precipitation rates here are related mostly to strongly enhanced AR moisture in these subregions, rather than changes in *ω*_700_. Last, UML precipitation histograms display near‐uniform increases in the sampling of all precipitation rates, reflecting the smallest change in mean precipitation rates here (Figure 10). Similarly, histograms of *ω*_700_ in the UML show an enhanced sampling across a broad range of vertical velocities, both more and less negative than the Baseline mode. Regardless, these changes skew more toward positive *ω*_700_ values, accounting for the apparent damping of vertical velocities here.

#### 3.5.3 Accounting for Increasing AR Area

We have discussed already that as SST increases in these experiments, so too does AR area. This enhancement in our sample size then makes it difficult to compare AR statistics across SST runs, particularly because means presented are obscured by an increased sampling of grid points farther from the narrow AR core which features the highest IVT and most intense oversea precipitation rates (Neiman et al., 2008). We first leveraged histograms of AR quantities to make comparisons despite unequal sample sizes, since that avoided issues associated with averaging AR core and periphery points together. Unfortunately, increasing AR area still makes histograms difficult to interpret, first because the upward shift can make left‐ or rightward shifts less obvious and second because it cannot explicitly filter for core or periphery AR points.

We first wish to determine the contribution of changing AR area on mean AR precipitation rate changes. To that end, we compare fractional changes in AR area (*AR*_*A*_; that is, the average combined area of all ARs in a given subregion) and AR area‐integrated precipitation (*P*_*A*_). Table 3 presents fractional changes of these quantities under SST increases.

**Table 3:** Fractional Changes of Mean Area‐Integrated AR Precipitation (*P*_*A*_) and AR Area Coverage (*AR*_*A*_) in Each Analysis Subregion (% K^−1^)

		$\frac{\Delta P_{A}}{P_{A}}$	$\frac{\Delta AR_{A}}{AR_{A}}$			$\frac{\Delta P_{A}}{P_{A}}$	$\frac{\Delta AR_{A}}{AR_{A}}$
LST	+2 K	2.77	10.64	UST	+2 K	5.97	5.78
	+4 K	14.82	20.41		+4 K	6.61	3.61
	+6 K	15.12	24.75		+6 K	6.47	3.55
LML	+2 K	13.00	12.19	UML	+2 K	34.25	37.23
	+4 K	12.38	11.81		+4 K	48.22	50.01
	+6 K	11.27	9.88		+6 K	50.42	54.83

We find that fractional increases in AR area are much larger than those in AR *P*_*A*_ in the LST, further supporting our hypothesis that decreases in mean AR precipitation rates here are related to enhanced AR areal coverage featuring weaker rates. The UST tells a different story however, with fractional changes in AR *P*_*A*_ exceeding those of AR area for all SST test runs, particularly the +4 K and +6 K scenarios. The precipitation distributions for this subregion (Figure 11b) show a marked increase in fractional area occupied by intense AR precipitation rates (≥70 mm/day) under higher SST conditions, allowing fractional increases in AR *P*_*A*_ to surpass those in AR area. ARs in the LML behave similarly, though fractional changes in AR *P*_*A*_ only just outpace those in AR area. Again, histograms of precipitation in this subregion (Figure 11c) provide additional context for these changes: Much of the increased areal extent of AR precipitation occurs at above‐average rates (≥30 mm/day), facilitating the observed slight acceleration of changes in AR *P*_*A*_ with respect to AR area. Finally, results in Table 3 for the UML reinforce expectations set up by the precipitation distributions (Figure 11d), wherein nearly equivalent changes in AR *P*_*A*_ and AR area are supported by a relatively uniform rise in the occurrence of all AR precipitation rates.

**Figure 12:**
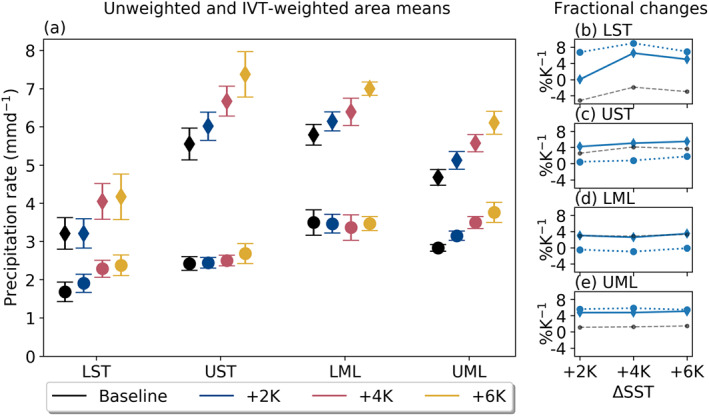
(a) Area‐mean precipitation rates for all SSTs (mm/day). Circles indicate unweighted means, while diamonds indicate that the means were weighted by IVT values, as described by Equation 5. Markers show the median value with respect to the 20‐member ensemble, while error bars show the interquartile range. Panels (b)–(e) show the fractional change in area precipitation means with respect to the Baseline for each uniform SST increase (% K^−1^). We use dotted lines for nonweighted and solid lines for IVT‐weighted, with line markers consistent with those used in panel (a). Gray, dashed lines show the same fractional changes in AR precipitation as in Figures 10c–10f for reference.

Second, we test the hypothesis that computed area‐mean AR precipitation changes (Figures 10c–10f) are influenced heavily by an increased sampling of weak, periphery AR points which naturally feature lower precipitation rates. We do this by comparing total precipitation rates against total precipitation rates weighted with IVT values. We compute these “IVT‐weighted” precipitation means (*P*_*IVT*_) like so

(5) $$P_{IVT}=\frac{\int_{A}P\cdot IVTdA}{\int_{A}IVTdA}$$

where *A* is the area of the subregion and *dA* is the grid point area. This approach has a twofold advantage: By weighting precipitation proportionally to IVT, it not only skews area means toward ARs without necessitating a binary AR filter but also weights the analysis toward the highest IVT points found in ARs. Therefore, we consider the *P*_*IVT*_ means shown in Figure 12 analogous to precipitation rates in the high‐IVT AR core. We thus compare fractional changes in them side‐by‐side with those computed directly for AR precipitation in Figure 12, in an effort to tease apart the effect of enhanced AR area on mean changes in AR precipitation rates.

In the LST in particular, we see a marked difference in the fractional changes of AR precipitation rates and *P*_*IVT*_: While the former decreases as SST increases, the latter shows no change under the +2 K scenario, and large increases in the +4 K and +6 K SST forcings of 6.54 and 5.00% K^−1^, respectively (Figure 12b). For the UST, increases in *P*_*IVT*_ only slightly outpace those in AR precipitation rates (Figure 12c); since AR area does not increase more than AR *P*_*A*_ here (Table 3), we would not expect substantially larger changes in *P*_*IVT*_ here either. Meanwhile, in the LML, changes in *P*_*IVT*_ track almost exactly with those in AR precipitation rates (Figure 12d), suggesting that increasing AR area does not play a large role in AR precipitation rate statistics in this subregion. Last, Figure 12e shows that *P*_*IVT*_ enhancement in the UML remains steady at approximately 5% K^−1^), providing a stark contrast to the mean AR precipitation rate increases of approximately 1% K^−1^) here. Overall, this analysis indicates that high‐IVT grid points such as those found in or near AR cores will feature regionally large enhancement in mean precipitation rates.

## 4 Conclusions

CAM5 was employed in its aquaplanet configuration (global, zonally‐symmetric, prescribed SSTs) with uniformly increased SSTs of +2K, +4K, and +6 K over the control “QOBS” profile (Neale et al., 2012). While certainly not a realistic model configuration or climate change signal, this experimental setup allowed us to isolate the impacts of increased SST on ARs, with the goal of evaluating fractional changes conditioned on SST of AR IVT, IWV, and precipitation rates, and validating the model response against more than a decade of theory. We detected ARs with an objective algorithm conditioned on finding local ridges in the IVT field at any given time step (i.e., the algorithm is insensitive to horizontal average increase in specific humidity). To facilitate simple regional comparisons, we divide our analysis domain into four subregions of approximately equal surface area.

We find that as SSTs increase so too does AR occurrence frequency, especially toward the higher latitudes in our test domain (Figure 2a). We attribute this domain‐wide enhancement in AR occurrence frequency largely to an overall increase in the size of ARs, (defined here as “zonal extent”; Figure 2b), a result which agrees with previous work using an independently developed ARDT (Espinoza et al., 2018). Meanwhile, the steady poleward movement of AR occurrence frequency maxima under SST warming is related to a poleward shift in EDJ position (Figure 3b), another result in agreement with existing AR literature (Gao et al., 2016; Mundhenk et al., 2016; Shields & Kiehl, 2016).

Alongside changes in AR occurrence and morphology, we also find changes in AR vapor transport statistics. In general, ARs experience super‐CC enhancement in IWV with respect to SST (from ∼6.3 to 9.7% K^−1^ depending on subregion and SST scenario; Figure 6), though the magnitude of this enhancement is in part a response to upper‐tropospheric temperature increases that outpace those in SST (Figure 7). Since the large enhancement of warming in the upper troposphere arises as a natural consequence of a dampened moist‐adiabatic lapse rate under surface warming conditions (e.g., Payne et al., 2020; Siler & Roe, 2014), we consider this result more reassuring than surprising. Similar rates of AR IWV enhancement have been observed in a coupled model (Gao et al., 2015) and shown to be related to enhancements in (a) CC sensitivity to below‐freezing temperatures in the upper troposphere and (b) latent heat release aloft as the atmosphere moistens (Payne et al., 2020). Meanwhile, we find that AR IVT also increases, though it occurs at a lower rate than IWV for all subregions (∼3.4 to 6.6% K^−1^; Table 1). We perform a very simplified analysis of the dynamical and thermodynamical contributions to AR IVT (Equation 2) and find that this result is related to a systematic decrease in AR low‐level winds speeds, which slow at fractional rates of ∼−0.8 to −5.5% K^−1^ (Table 1).

Taken together, the relative changes in AR IWV and IVT are comparable to those seen in other studies (Gao et al., 2015; Warner et al., 2014) as is the finding that AR IVT will increase mostly due to thermodynamical effects (enhanced AR IWV) rather than dynamical ones (AR low‐level winds) (Dettinger, 2011; Gao et al., 2015, 2016; Lavers et al., 2013; Warner et al., 2014). Dynamical changes are further observed in changing patterns of AR characteristics. Specifically, a poleward‐shifting EDJ (Figure 3b) pushes ARs poleward with the storm track (Figure 2a), a result which is documented in comprehensive climate models (Gao et al., 2016; Shields & Kiehl, 2016). We also find an apparent connection between the STJ and AR vIVT characterized by a fractional enhancement of AR vIVT on the cyclonic side of the STJ, and a suppression of AR vIVT on its anticyclonic side (Figures 5g–5h). Although the STJ shows a discontinuous response to SST increase (Figure 3b), the result suggests a relatively easily diagnosed large‐scale dynamical control on potential moisture sources for ARs.

Despite systematic increases in AR IWV and IVT, AR precipitation has a much more varied response across latitudes (∼−5.2 to 4.1% K^−1^; Figure 10). Similar to our analysis on AR IVT, we perform a simplified calculation derived from theory to contextualize these changes in terms of dynamical and thermodynamical contributions (Equation 4) and generally find a compensatory relationship characterized by consistent increases in moisture (in this case, near‐surface *q*) and a systematic damping in midtroposphere vertical velocities (Table 2). An examination of precipitation distributions provides some important context for these findings: Across all analysis subregions, the highest precipitation rates always increase, hinting at a robust enhancement in AR extreme precipitation even in areas where mean AR precipitation decreases (Figures  11a–11d). Further complicating our results on mean precipitation rates is the finding that AR area increases as SST does (Figure 2b and Table 3). While AR area increases have been noted before (Espinoza et al., 2018), the enhanced AR area can nevertheless obscure interpretation of ARDT‐derived conclusions about mean AR characteristics. Here, a simple analysis on AR area and precipitation (section 3.5.3) shows that by sampling more grid points toward the weaker periphery of AR objects, fractional changes in zonal mean AR precipitation rates are damped in three out of four analysis subregions (Figure 12). Nonetheless, a clear enhancement in precipitation rates within ARs is observed in the UST and LML analysis subregions (Figures 12b and 12c).

We approached this study with the goal of leveraging an idealized model to investigate the sensitivity of AR statistics to isolated SST warming. In fact, the idealizations present in this experiment design provide a means to more clearly elucidate the underlying physical processes and explain the relevant theory. The analyses of AR IWV and precipitation described here provide physical context for results obtained in comprehensive climate models, where compensatory or compound effects can confound interpretation and leave open questions about the mechanistic relationships between forcings and impacts.

## Supporting information



Text S1

## Data Availability

Previous and current CESM versions are freely available online (at https://www.cesm.ucar.edu/models/cesm2/). TempestExtremes AR detection software are available for public use on GitHub (at https://www.github.com/ClimateGlobalChange/tempestextremes). Data used to generate figures for this manuscript, as well as model configuration and AR detection details, are archived at Zenodo (https://doi.org/10.5281/zenodo.3912050).
