# Supplementary material for: Sensitivity of Atmospheric River Vapor Transport and Precipitation to Uniform Sea Surface Temperature Increases
Source: J Geophys Res Atmos. 2020 Oct 29;125(21):e2020JD033421. doi: 10.1029/2020JD033421 (PMC7771035; doi:10.1029/2020JD033421)
Supplement: Supplementary file 1 — Text S1 [file JGRD-125-e2020JD033421-s001.pdf]

Supporting Information for "Sensitivity of atmospheric river vapor transport and precipitation to uniform sea-surface temperature increases"

Elizabeth E. McClenny[1], Paul A. Ullrich[1], Richard Grotjahn[1]

[1]Atmospheric Science, University of California, Davis, Davis, California, United States

Contents of this file

1. Text S1

2. Figures S1 to S7

Text S1

In this section we examine the sensitivity of the AR width to the Laplacian criteria under a multiplicative enhancement in total IVT. We begin by considering an idealized atmospheric river with Gaussian cross-section in IVT,

$$IVT(s) = IVT0 + dIVT \exp(-s^2/w^2), \tag{1}$$

where $IVT(s)$ is the pointwise IVT at distance s meters along the cross-section, $IVT0$ is the constant background IVT, $dIVT$ is the anomalous IVT enhancement from the AR, and w is the e-folding width of the AR. Since $IVT0$ is unimportant to the Laplacian criteria, we set $IVT0 = 0$ in this analysis. Figure S2 depicts two such Gaussian profiles with e-folding width $w = \pi/90$ rad $= 222$ km, and a baseline $dIVT = 500$ kg/m/s and

$dIVT = 500 \times 1.07^6 \approx 750$ kg/m/s, the latter corresponding to a 7% increase in IVT per degree C under a +6K experiment.

The second derivative of (1) with respect to s, which is equivalent to the Laplacian for an AR with no variation perpendicular to the cross-section, is then given by

$$\frac{d^2IVT}{ds^2}(s) = \frac{dIVT(4s^2 - 2w^2)}{w^4} \exp(-s^2/w^2), \tag{2}$$

with units of kg/m/s/m^2. To convert to kg/m/s/rad^2, which is used in our ARDT, we multiply by $(6.37122 \times 10^6 \text{ m/rad})^2$. The resulting profiles of the Laplacian are depicted in Figure S2 along with the employed threshold of -40000 kg/m/s/rad^2. As can be seen in this figure the number of points satisfying the Laplacian threshold – that is, those points where the curve is below the dotted line – does not significantly change even when the strength of the AR is enhanced by 50%. To get a better handle on the magnitude of this change we can solve numerically for the point at which the second derivative hits our threshold and find that this occurs at $s = 5263$ m for the baseline AR and $s = 5335$ m for the +6K AR. Thus the multiplicative enhancement results in a mere 1.4% increase in the AR width.

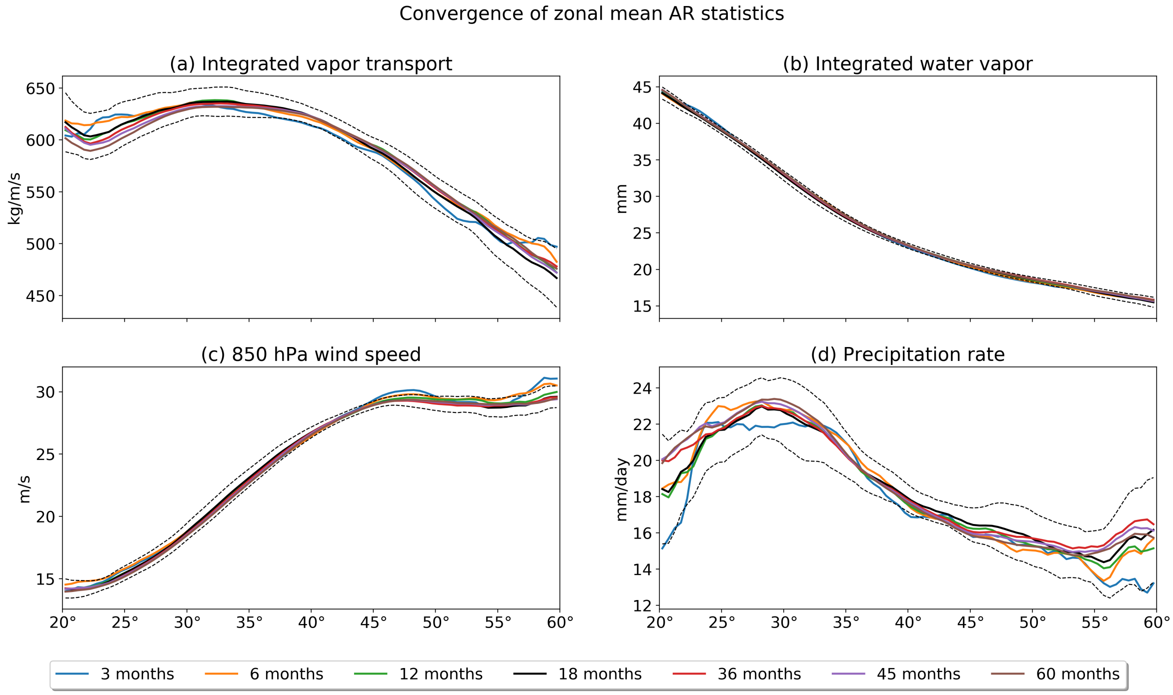

Figure S1. Zonal mean AR quantities for the "Baseline" SST run. Line colors indicate the number of months in the sample. Black dashes show the 18-month mean +/- one standard deviation with respect to the full ensemble.

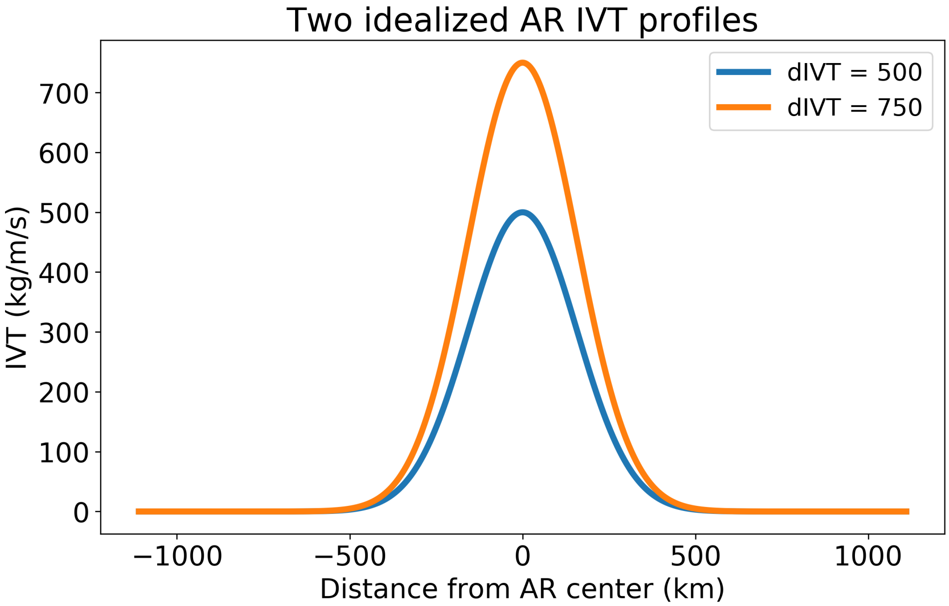

Figure S2. A depiction of the idealized Gaussian cross-sections used in this analysis with $dIVT = 500$ kg/m/s and $dIVT = 750$ kg/m/s, which represent typical ARs under baseline and +6K experiments.

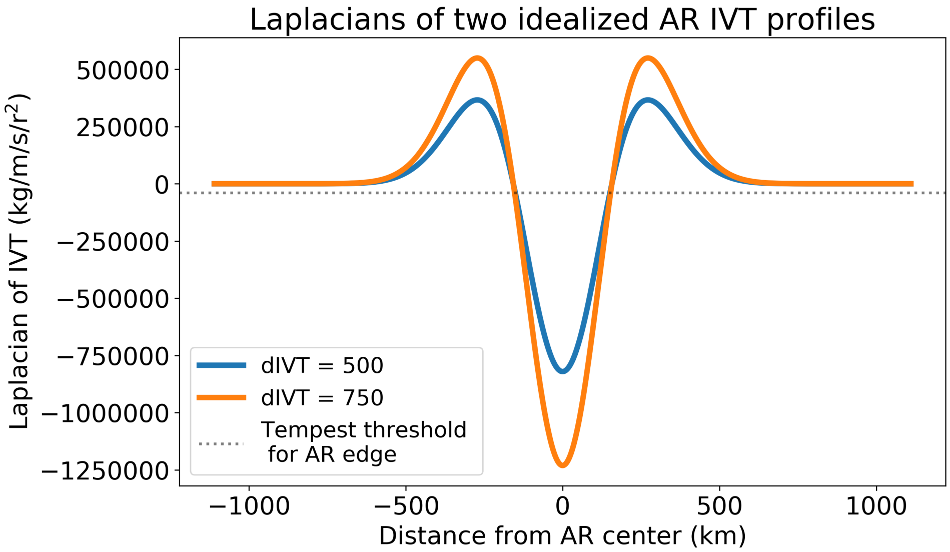

Figure S3. As in Figure S2, except depicting the second derivatives of the Gaussian cross-sections.

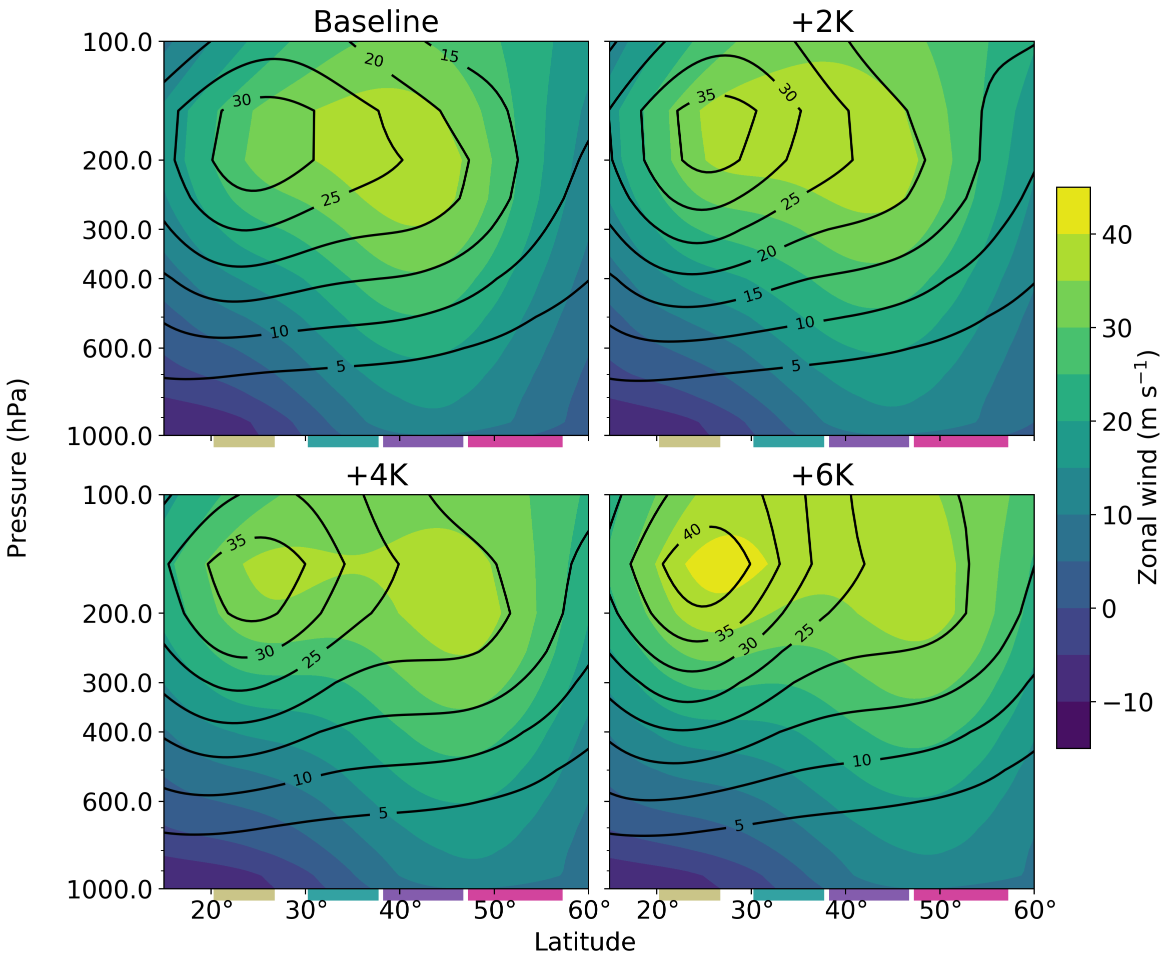

Figure S4. Zonal jets for each SST run. Filled contours show zonal-mean zonal wind (m/s); the eddy-driven jet can be seen extending through the troposphere in the midlatitudes. Unfilled contours show zonal-mean zonal wind minus 850 hPa zonal wind; the upper-tropospheric maximum seen in each panel is the subtropical jet. Colored boxes and labels on x-axis denote analysis subregions described in the main text (Section 2.4).

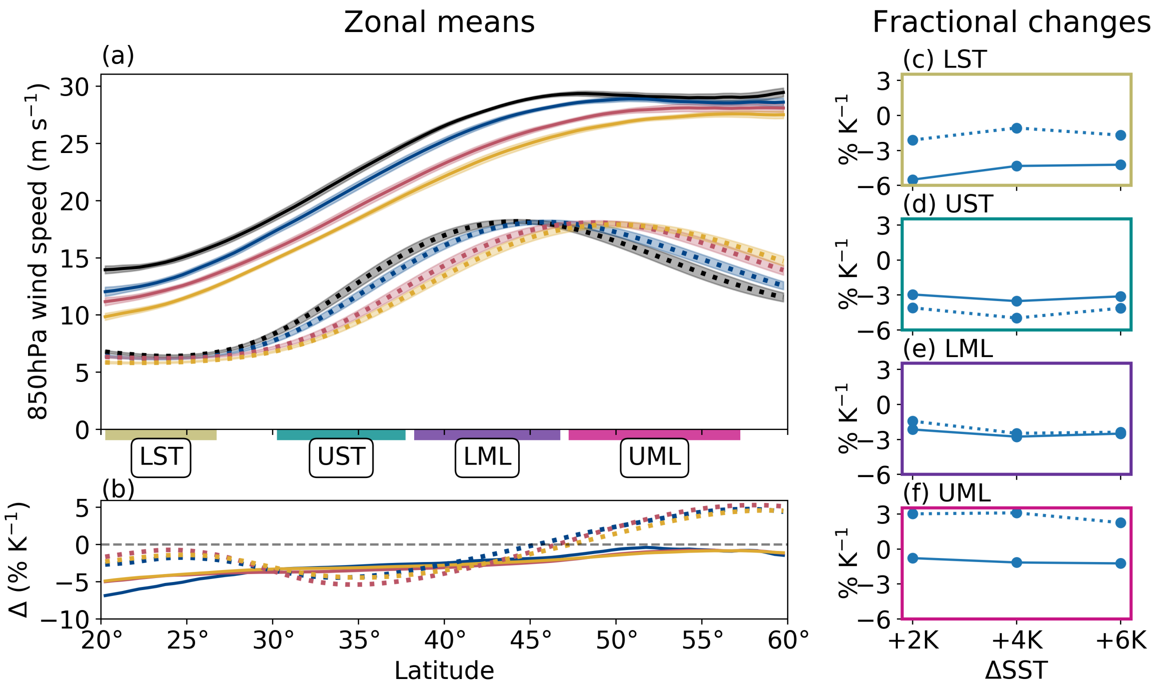

Figure S5. (a) Meridional distributions of zonal mean AR (solid) and non-AR (dotted) 850 hPa wind speed. Shading shows 95% confidence intervals. (b) Relative differences with respect to the Baseline SST (%/K), using the same line color and style conventions. (c-f) Area-weighted mean relative change per K SST increase (blue; line style conventions as before). Colored boxes and labels on x-axis denote analysis subregions described in the main text (Section 2.4).

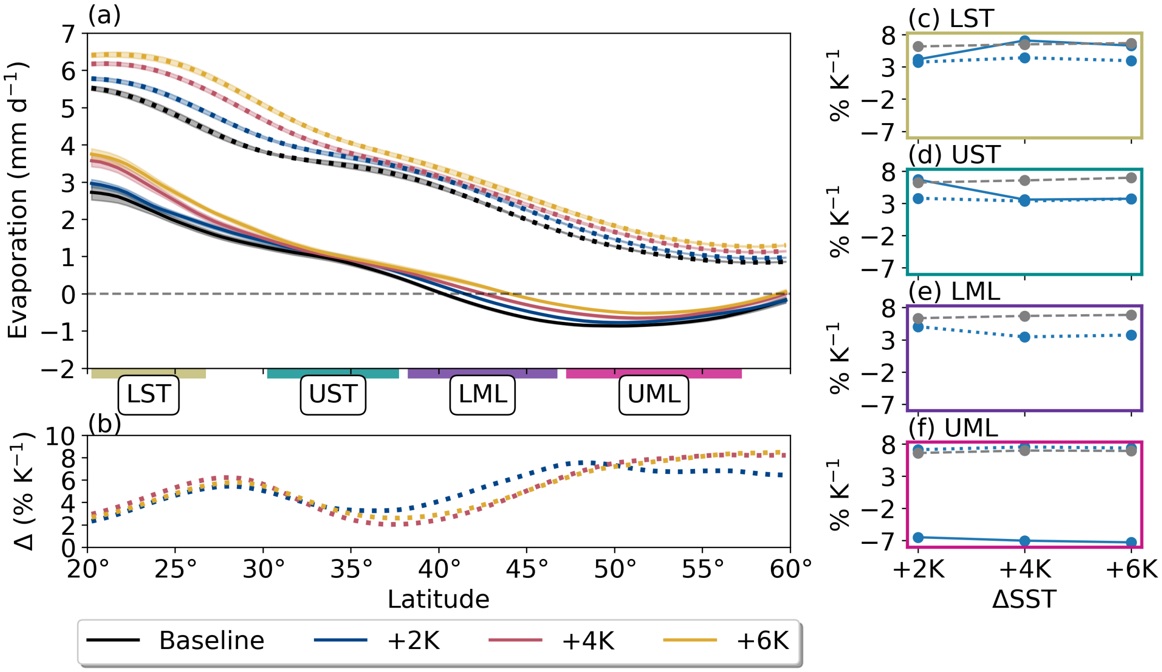

Figure S6. As Figure S5, but for surface evaporative flux, and with the addition of Clausius-Clapeyron predictions for near-surface saturation vapor pressure for reference (grey, dashed lines in c-f). Note numerical issues prevented us from plotting fractional changes in AR surface evaporative flux: since AR evaporation is near-zero in the LML, fractional changes through this region were artificially inflated.

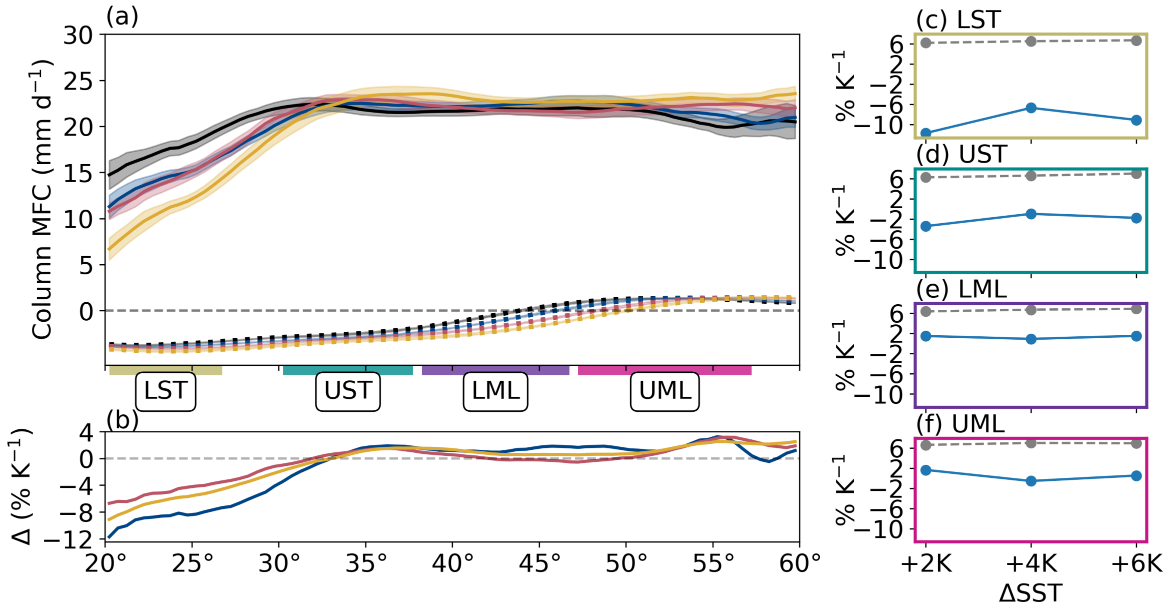

Figure S7. As Figure S6, but for column-integrated moisture flux convergence (MFC). Fractional changes are not shown for non-AR MFC since its very small magnitudes tended to result in spuriously large values.
